# Aerobic Exercise Training Increases Circulating sRAGE in Adults With Type 2 Diabetes: Associations With Sheddase Regulation

**DOI:** 10.1111/dom.70995

**Published:** 2026-06-17

**Authors:** Ryan K. Perkins, Corey E. Mazo, James Shadiow, Pallavi Varshney, Edwin R. Miranda, Victoria R. Miranda, Maggie Eisenberg, Jane E. Nakamura, Elif A. Oral, Jacob M. Haus

**Affiliations:** ^1^ Kinesiology Department California State University Chico California USA; ^2^ School of Kinesiology University of Michigan Ann Arbor Michigan USA; ^3^ Department of Surgery University of Michigan Ann Arbor Michigan USA; ^4^ Department of Nutrition and Integrative Physiology University of Utah Salt Lake City Utah USA; ^5^ Department of Health and Kinesiology University of Utah Salt Lake City Utah USA; ^6^ Department of Internal Medicine—Metabolism Endocrinology, and Diabetes University of Michigan Medicine Ann Arbor Michigan USA

**Keywords:** aerobic exercise, diabetes, inflammation, soluble receptor for advanced glycation endproducts, soluble toll like receptor

## Abstract

**Objective:**

Soluble receptor for advanced glycation end‐products (sRAGE) and soluble Toll‐like receptor 4 (sTLR4) are circulating pattern recognition receptor isoforms implicated in inflammatory regulation in type 2 diabetes mellitus (T2DM). This study examined the effects of supervised aerobic exercise training (AET) on circulating sRAGE, sTLR4, and related skeletal muscle inflammatory signalling outcomes in adults with T2DM.

**Methods:**

Thirty‐three sedentary adults with T2DM were randomized to AET (*n* = 20; age: 54.5 ± 9.3 years; BMI: 34.6 ± 1.3 kg/m^2^; HbA1c: 7.5% ± 1.1%) or standard of care control (CON; *n* = 13; age: 59.6 ± 9.3 years; BMI: 32.6 ± 1.6 kg/m^2^; HbA1c: 6.9% ± 0.9%). Participants in AET completed 12 weeks of supervised aerobic exercise (60 min/day, 5 days/week) at ~70% VO2peak. Pre—and post‐intervention assessments included oral glucose tolerance testing, DXA‐derived body composition, VO2peak testing, and an acute aerobic exercise trial with blood and skeletal muscle sampling. Circulating sRAGE, sTLR4, hsCRP, and skeletal muscle protein expression of RAGE, TLR2/4, ADAM10, MMP2/9, and TIMP1/3 were quantified.

**Results:**

AET increased circulating sRAGE concentrations across the intervention period, whereas sTLR4 was unchanged. AET improved cardiorespiratory fitness, reduced body fat percentage and fat mass, lowered fructosamine concentrations, and increased the Matsuda Index. In skeletal muscle, significant alterations were observed in selected TIMP:MMP and TIMP:ADAM10 ratios, although no changes were detected in ADAM10 activity or skeletal muscle RAGE protein expression. Exploratory analyses demonstrated associations between changes in circulating sRAGE and aerobic fitness.

**Conclusions:**

AET increased circulating sRAGE and improved select metabolic health outcomes in adults with T2DM. These findings are consistent with the concept that chronic exercise training may favourably influence inflammatory regulation in metabolic disease. However, the tissue‐specific mechanisms contributing to exercise‐induced changes in circulating sRAGE remain incompletely understood and warrant further investigation.

## 1 Introduction

Type 2 diabetes mellitus (T2DM) is a chronic metabolic disorder characterized by dysregulated glucose metabolism, leading to an increased risk of cardiovascular disease, kidney dysfunction, and other comorbidities [1]. Lifestyle interventions, particularly aerobic exercise training, have demonstrated benefits for improving glycemic control and reducing the risk of complications associated with T2DM [2, 3, 4, 5] In parallel, conserved inflammatory signalling pathways mediated by pattern recognition receptors (PRRs), including the receptor for advanced glycation end‐products (RAGE) and Toll‐like receptors (TLRs), have emerged as key contributors to T2DM pathophysiology [6]. RAGE, a multiligand PRR, is implicated in the cellular response to advanced glycation end‐products (AGEs) and other pathogen—and damage‐associated molecular patterns (PAMPs and DAMPs, respectively), perpetuating the inflammatory environment characteristic of T2DM. Similar to RAGE, TLR2 and TLR4 play fundamental roles in the innate immune response due to their overlap in activation by PAMPs and DAMPs, typically elevated in those with metabolic disease and convergence on downstream signalling mechanisms [7, 8, 9, 10]. Of the 10 TLRs in human skeletal muscle, TLR2 and TLR4 are among the most highly expressed [11, 12] and are also upregulated in those with metabolic dysfunction [13, 14, 15]. It is through the inflammatory link, at least in part, that genetic deletion and blockade of RAGE [16, 17] and TLR [18, 19, 20, 21] signalling improves metabolic function, supporting a mechanistic link between PRR signalling and metabolic regulation.

Soluble RAGE (sRAGE), generated through proteolytic cleavage or alternative splicing, has been proposed to act as a decoy receptor that binds circulating ligands and limits RAGE‐mediated signalling. Lower circulating sRAGE concentrations are commonly observed in individuals with obesity and impaired glucose tolerance and are often inversely associated with indices of metabolic dysfunction [22, 23, 24, 25, 26, 27, 28]. However, the functional role of sRAGE in glucose regulation remains incompletely defined. It is not yet clear whether sRAGE primarily reflects improved insulin sensitivity, altered insulin secretion, or broader changes in glycemic control, and disentangling these relationships requires well‐controlled human intervention studies.

Extracellular matrix sheddases, including matrix metalloproteinases (MMPs) and a disintegrin and metalloproteinase 10 (ADAM10), along with their endogenous inhibitors (TIMPs), are established regulators of tissue remodelling and receptor ectodomain shedding [29, 30, 31, 32, 33]. These enzymes can cleave membrane‐bound PRRs, releasing soluble receptor isoforms into circulation. Prior mechanistic work supports a role for ADAM10 and select MMPs in RAGE shedding, with regulation linked to calcium‐dependent signalling pathways and cellular stress responses [32, 33, 34, 35]. Given that AE induces robust calcium flux, redox‐sensitive signalling, and tissue remodelling, it provides a physiologically relevant context to examine regulation of these pathways. However, whether exercise‐induced changes in circulating sRAGE are mediated by alterations in sheddase activity remains a working hypothesis rather than an established mechanism.

Importantly, the effect of exercise on circulating sRAGE is not consistent across studies. While several reports demonstrate increases in sRAGE following AE [7, 36, 37, 38, 39, 40, 41, 42], others have observed reductions or no change depending on the population studied (e.g., obesity, chronic kidney disease, or differing exercise stimuli) [39, 43, 44]. These discrepancies suggest that disease context, baseline metabolic status, and exercise characteristics may critically influence the sRAGE response, underscoring the need for controlled trials in well‐characterized populations.

Skeletal muscle represents a biologically plausible, but not exclusive, tissue of interest in this context. As a primary site engaged during AE, muscle contraction induces calcium signalling, activates redox‐sensitive transcriptional pathways, and promotes tissue remodelling processes that could influence sheddase activity and PRR regulation [13, 14, 15, 16, 17, 19, 20, 21, 45]. Thus, AE provides a unique In vivo model to interrogate coordinated changes in circulating and tissue‐specific inflammatory signalling, while acknowledging that multiple tissues may contribute to circulating sRAGE.

Accordingly, this study aimed to determine the impact of a supervised 12‐weeks AE intervention on circulating sRAGE and sTLR4, as well as their regulatory components, in individuals with T2DM. We additionally examined responses to an acute AE bout to assess dynamic regulation of circulating and skeletal muscle inflammatory signalling involving RAGE, TLR2, and TLR4. We hypothesized that AE training would increase circulating sRAGE and that these changes would be associated with alterations in key sheddases (MMPs and ADAM10) and their TIMP regulators.

## 2 Materials and Methods

### 2.1 Participants

Female and male participants between the ages of 40–75 years were recruited from the greater Ann Arbor, MI area through multiple means, including physical flyers, social media, University of Michigan Medicine research databases, and word‐of‐mouth. Inclusion criteria consisted of current T2DM diagnosis, BMI of 26–44 kg/m^2^, and sedentary behaviour (< 30 min of exercise, 3 times per week). Exclusion criteria included history of cardiovascular, cerebrovascular, renal or haematological disease, age < 40 or > 75 years, current tobacco use, and currently pregnant individuals. All participants provided informed, written consent before participation. This study was approved by the Institutional Review Board at the University of Michigan (#HUM00146001) and is registered at ClinicalTrials.gov (NCT03534687).

### 2.2 Study Design

#### 2.2.1 Randomization and Experimental Design

**FIGURE 1:**
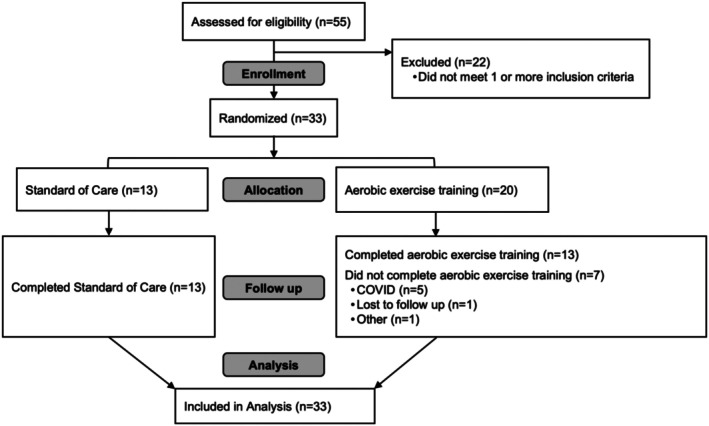
CONSORT Diagram. A total of 55 males and females underwent screening based on inclusion and exclusion criteria. Following screening, 33 participants were eligible to enrol in the 12‐weeks intervention. Participants were randomized to an aerobic exercise training (AET; *n* = 20) or standard of care control group (CON; *n* = 13). Seven participants did not complete the AE training period. Final analysis was performed on 33 participants.

This study consisted of four study visits completed by all participants before and repeated after the intervention period. Briefly, visits included (1) consenting, screening, and oral glucose tolerance test (OGTT), (2) DXA body composition assessment, (3) VO2peak treadmill testing, and (4) acute AE treadmill trial. After completing physical activity and medical health history questionnaires during screening to determine eligibility, participants were randomized to a control (CON) or aerobic exercise training (AET) group (Figure 1) between February 2019 and May 2023. Prior to enrollment, all participants underwent comprehensive safety screening procedures. This included a detailed medical history assessment, blood and urine laboratory testing (including complete blood count, metabolic panel, HbA1c, lipid profile, and renal function), and a resting 12‐lead electrocardiogram. Participants also completed a graded exercise stress test with VO2peak measurement under clinical supervision. All clinical results were reviewed by a study physician, and any clinically significant findings resulted in referral to the participant's personal physician and exclusion or delayed enrollment until medically cleared. These procedures ensured that participants were medically stable and safe to undergo exercise testing and training.

Randomization to AET or CON was performed by a member of the research team with no clinical involvement in the trial. Participants randomized to CON were provided the opportunity to re‐enrol into the AET intervention upon completion of their original CON assignment. The intent of this reenrollment option was to provide equitable access to the intervention hypothesized to improve study outcomes and maximize adherence to preexisting lifestyle habits during the trial period, thereby reducing potential confounding factors.

A total of 55 participants expressed interest in the study, of which 22 were excluded due to inclusion and/or exclusion criteria (Figure 1). Therefore, 33 participants were randomized to either CON (*n* = 13; 69% female) or AET (*n* = 20; 70% female). Eight participants (75% female) originally randomized to CON elected to reenroll in AET after completing their original group allocation. In total, 7 participants did not complete the AET intervention period (i.e., lost‐to‐follow up, COVID logistical complications, or illness). Thirty‐three participants were randomized (AET *n* = 20, CON *n* = 13). Linear mixed‐effects models included all available observations under maximum likelihood estimation. Completion rates for individual outcomes varied due to biopsy availability and attrition.

Participants who re‐enrolled into the AET intervention completed repeat baseline testing and were treated as AET observations. To account for repeated measures within individuals, participant ID was included as a random effect in all linear mixed‐effects models. In addition, sensitivity analyses excluding re‐enrolled participants were performed for primary outcomes to assess robustness of findings.

All participants were provided with the American Diabetes Association Standard of Care for diabetes management [46] and were instructed to maintain current physical activity or diet habits outside of study‐related activities. Participants in the CON group were not subject to continuous or device‐based physical activity monitoring; instead, changes in cardiorespiratory fitness (VO_2_peak) from baseline to follow‐up were used as a physiological indicator of adherence to habitual activity levels. No device‐based monitoring was used; however, stability of VO_2_peak across time supported adherence to habitual activity levels.

**FIGURE 2:**
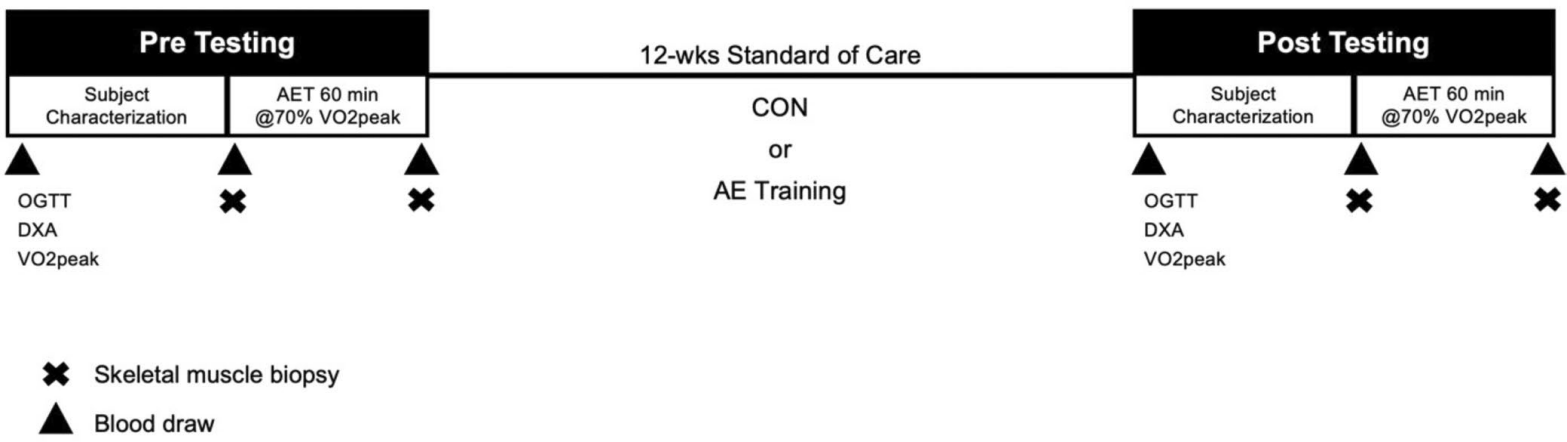
Prior to group randomization (CON, control; AET, aerobic exercise training), participants underwent body composition assessing (DXA), oral glucose tolerance testing (OGTT), and a VO2peak cardiovascular fitness test. Within 48 h of randomization, participants underwent a pre‐intervention acute exercise trial (acute AE) for 60 min at 70% VO2peak, with vastus lateralis skeletal muscle biopsies and blood draws taken before and 30 min post‐acute AE. Following this pre‐testing period, the CON group received 12 weeks of standard of care, while the AET group received 12 weeks Standard of Care and supervised aerobic exercise training. DXA, OGTT, VO2peak testing, and the AET were repeated following the 12‐weeks intervention period.

Individuals randomized to AET participated in 12 weeks of supervised training sessions, consisting of treadmill, stationary cycle ergometer, elliptical, and/or upper‐arm ergometry exercise (Figure 2). Treadmill exercise was the primary modality, with alternative modes used on an as‐needed basis (e.g., musculoskeletal discomfort) while maintaining prescribed intensity and duration targets. Initial VO2peak testing (Visit 3; treadmill‐based graded exercise test) was conducted for all participants. HR was monitored continuously during training sessions (Polar, Kempele, Finland) and intensity was adjusted to reach a HR that corresponded to the target % of VO2peak. Participants in the AET group exercised 5 days/week, with each session incorporating a short warmup and cooldown during training sessions to reach a total target duration of 60 min. The training program started with a 2‐week ramp‐up, familiarization period of progressing exercise intensity and duration. Exercise intensity began at a target intensity of 55% VO2peak for week 1 (40 min/session), progressing to 60%–65% VO2peak for week 2 (50 min/session), and 70% VO2peak for all other weeks (60 min/session). Follow‐up VO2peak tests were performed at weeks 4 and 8 to monitor progress and AE intensity was adjusted to match prescribed training HR [47]. Session workloads (e.g., speed, grade, or resistance) were adjusted in real time to maintain HR within target zones, and target HR ranges were recalibrated following repeat VO2peak testing at weeks 4 and 8 to account for training adaptations. AE sessions were supervised by at least one member of the research team and all participants in AE completed > 90% of exercise training sessions. Adherence to the exercise prescription (target intensity and duration) was monitored during each session using continuous HR tracking.

Blinding of participants and interventionists was not feasible due to the nature of the exercise intervention. However, steps were taken to minimize bias in outcome assessment. All biospecimens were de‐identified and coded prior to laboratory analysis, and investigators performing biochemical assays and data generation were blinded to group allocation and intervention status. Data analysis was conducted using coded group identifiers, with unblinding occurring only after primary analyses were completed. Outcome measurements were performed by trained laboratory personnel who were not involved in participant randomization or intervention delivery.

#### 2.2.2 Metabolic Control

For assessments sensitive to diet, medication, and physical activity, standard metabolic controls were closely monitored. More specifically, before the OGTT and acute AE, participants were asked to refrain from eating for ~10 h (overnight fast), withhold oral antidiabetic medications the morning of testing, not consume alcohol for 48 h, and abstain from vigorous physical activity and caffeine consumption for 24 h prior to testing. Medication use was recorded and monitored throughout the study. Participants were instructed to withhold oral anti‐hyperglycemic medications on the morning of testing. For injectable GLP‐1 receptor agonists, testing was scheduled to align with dosing intervals. Participants using basal insulin were instructed to withhold morning doses prior to testing when applicable. Dietary intake was recorded for 3 days prior to testing using ASA24, and participants were instructed to replicate their pre‐intervention diet and consume approximately 250 g of carbohydrate per day to minimize variability in glycogen status. To account for blood volume shifting that occurs with changes in body position or engagement in exercise [48], participants rested quietly (seated) for 30 min prior to all blood draws.

#### 2.2.3 Oral Glucose Tolerance Test and Calculations of Metabolic Indices

A 75‐g oral glucose tolerance test (OGTT) was performed at baseline and following the intervention, 48 h after the final exercise bout. Briefly, an antecubital or dorsal hand venous catheter was inserted, and fasting blood samples were obtained. Participants then consumed 75 g anhydrous glucose dissolved in 10 oz. of water within ~3 min. Venous blood samples were subsequently collected at 30, 60, 90, and 120 min post‐ingestion. Blood glucose was measured at bedside in duplicate using the Contour Next One Blood Glucose Meter and test strips (Ascensia Diabetes Care, Parsippany, NJ, USA), and a subset of samples was cross validated against a clinical automated laboratory platform to confirm accuracy. Incremental area under the curve (iAUC) for glucose, insulin, C‐peptide, and non‐esterified fatty acids (NEFA) was calculated using the trapezoidal method, with fasting values subtracted. Insulin resistance was estimated using the homeostatic model assessment of insulin resistance (HOMA‐IR) as: HOMA‐IR = [fasting insulin (μU/mL) × fasting glucose (mmol/L)]/22.5 [49]. Whole‐body insulin sensitivity was estimated using the Matsuda index: 10 000/√[(fasting glucose × fasting insulin) × (mean glucose × mean insulin)] [50]. Early‐phase *β*‐cell secretory response was estimated using the C‐peptidogenic index, calculated as the increment in C‐peptide from 0 to 30 min divided by the increment in glucose from 0 to 30 min during the OGTT. The oral disposition index was calculated as the product of the Matsuda Index and the C‐peptidogenic index [51].

#### 2.2.4 DXA Body Composition

Height and weight were measured via stadiometer and digital scale (InBody 770; InBody Co. Ltd., Seoul, Korea) respectively. Body composition was assessed via dual X‐ray absorptiometry (DXA; Lunar iDXA; GE Healthcare, Madison, WI).

#### 2.2.5 VO2peak Testing

Peak oxygen consumption (VO2peak) was determined using a Cornell treadmill protocol until participants reached volitional fatigue [52]. Due to clinical population considerations, VO2peak was used rather than requiring strict VO2max criteria in all participants. Expired air was analysed via indirect calorimetry (COSMED). HR was continuously monitored via 12‐lead electrocardiogram, and participants provided a rating of perceived exertion (RPE) at the end of each 2‐min stage. VO2peak was achieved in participants that met at least two of the five following criteria: volitional fatigue, a plateau in VO2 despite an increasing workload, RPE > 17, RER > 1.1, or a heart rate > 85% age‐predicted maximal HR.

#### 2.2.6 Acute Aerobic Exercise Trial

All participants completed a supervised acute AE trial before and after the 12‐weeks intervention. This design allowed differentiation between acute exercise responses and chronic training adaptations in circulating and skeletal muscle outcomes. All post‐intervention acute exercise trials were performed 48 h after the final training session to minimize residual effects of prior exercise. Upon arrival, participants rested quietly for 30 min (seated), after which a blood draw (antecubital vein) and *m. vastus lateralis* (VL) skeletal muscle biopsy was performed. The acute exercise session consisted of 60 min treadmill walking at a HR that corresponded with ~70% of pretraining VO2peak. VO2 was monitored for 5 min in 15 min intervals and speed and/or incline was adjusted to maintain target VO2 (Parvo Medics, Sandy, UT). Following the acute AE trial, participants rested quietly for 30 min, after which a final blood draw and VL biopsy was performed. This exercise bout was selected to provide a robust metabolic and cardiovascular challenge that aligns with American College of Sports Medicine and Physical Activity Guidelines for Americans [53, 54].

#### 2.2.7 Blood Sample Collection

All venous blood samples were collected into EDTA plasma, lithium‐heparin plasma, and serum SST vacutainers. Aprotinin and DPP‐4 inhibitors were added where appropriate to preserve analyte stability. For serum, whole blood was collected in SST vacutainers allowed to clot at room temperature for 30 min and was subsequently centrifuged at 3500 RPM for 10 min at 20°C. For plasma, blood was centrifuged at 3000 RPM for 10 min at 4°C. Following centrifugation, serum and plasma were stored at −80°C until analysis.

#### 2.2.8 Skeletal Muscle Biopsy

Skeletal muscle biopsies were taken from the VL before and 30 min after the acute AE trial. Pre—and postexercise biopsies were alternated between left and right leg. Biopsy sites were alternated between legs to minimize local tissue effects and repeated sampling‐related inflammation. Before the biopsy, local anaesthetic (2% lidocaine HCL) was administered followed by a small incision (~0.5 cm) at the biopsy site [41, 55, 56]. A Bergstrom biopsy needle was inserted with suction, extracting ~200 mg of muscle tissue. Muscle tissue was cleared of all visible connective tissue and fat, blotted with gauze to remove blood, immediately flash frozen in liquid nitrogen, and stored at −80°C until further analysis.

#### 2.2.9 Circulating Metabolites and Inflammatory Factors

All analytes and standards were measured in triplicate. Insulin (90 095, Crystal Chem, Elk Grove Village, IL) and c‐peptide (DICP00, R&D Systems, Minneapolis, MN) were assessed in lithium‐heparin collected plasma via enzyme‐linked immunosorbent assays (ELISA). Plasma fructosamine levels were determined via glycated serum protein assay (DZ112B‐KY1, Diazyme, Poway, CA). Experimental procedures were conducted following manufacturers' recommendations using calibrator (DZ112B‐CAL, Diazyme), control (DZ112B‐CON, Diazyme), or plasma samples. Average intra‐assay variability was 4.3% and ranged from 2.7%–6.8%. Nonesterified fatty acids (NEFA) were measured from EDTA plasma samples via enzymatic assay (Wako HR series, Fujifilm, Lexington, MA). sRAGE was quantified in EDTA plasma via sRAGE ELISA (DRG000, R&D Systems, Minneapolis, MN) according to manufacturer's instructions. The average inter‐assay and intra‐assay variability was 3.3% and 2.5%–3.9%, respectively. sTLR4 (MBS167606, My Biosource, San Deigo, CA) was assessed in EDTA plasma via ELISA. hsCRP was determined from serum via ELISA (#80955, Crystal Chem, Elk Grove Village, IL). Plates were read using SpectraMax ID5 Microplate Reader (Molecular Devices).

#### 2.2.10 Skeletal Muscle Protein Quantification and Enzymatic Activity Assay

Protein expression in skeletal muscle was quantified via Western blot analysis. Approximately 20 mg (wet weight) of frozen muscle tissue was homogenized by ceramic beads (lysing matrix D, FastPrep‐24 homogenizer, MP Biomedical, Santa Ana, CA) in 20 volumes of ice‐cold 1X Cell Lysis buffer (#9803, Cell Signalling Technology, Danvers, MA), 1X Protease Phosphatase Inhibitor (#5872, Cell Signalling Technology, Danvers, MA), and 25 μM‐MG132 (F1100, UBPBio, Dallas, TX). Total protein concentration was determined via BCA assay (Pierce Biotechnology, Rockford, IL). Equal protein (25 μg) was loaded on a 10% pre‐cast Criterion TGX (BioRad, Hercules, CA) and resolved using SDS‐PAGE, transferred to a nitrocellulose membrane, and blocked with Odyssey Blocking Buffer (LI‐COR Biosciences, Lincoln, NE) in TBS for 1.5 h at room temperature. Primary antibody (Table S2) diluted in blocking buffer containing 0.1% Tween 20 incubation took place overnight, at 4°C, with gentle rocking. After overnight incubation, membranes were washed 5 times with 1X TBST at room temperature for 5 min with vigorous shaking. Fluorophore‐conjugated secondary antibodies were prepared at 1:10 000 dilution in blocking buffer containing 0.1% Tween 20, and membranes were incubated in the dark for 1 h at room temperature. Secondary antibody incubations membranes were followed by 5 consecutive 1X TBST washes at room temperature for 5 min with vigorous shaking. Immunofluorescence was visualized with a NIR system (Odyssey, LICOR Biosciences, Lincoln, NE) and quantified using LI‐COR Image studio software v 5.2 (LICOR Biosciences, Lincoln, NE). Following protein of interest quantification, membranes were stained with Revert total protein stain for normalization.

ADAM10 activity was determined using a fluorometric ADAM10 activity assay (AS‐72226, Anaspec, Freemont, CA). Muscle samples were homogenized in ice cold assay buffer according to the manufacturer's instructions and 50 μg of protein was analysed in duplicate (3915, Corning‐Costar). Kinetic fluorescence was measured every 5 min for 60 min using the SpectraMax ID5 Microplate Reader (Molecular Devices, Sunnyvale, CA).

#### 2.2.11 Statistical Analysis

The primary outcome of the study was circulating sRAGE. Secondary outcomes were considered supportive and exploratory. Statistical analyses were performed using R (version 4.5.2; R Foundation for Statistical Computing, Vienna, Austria). Normality of baseline variables was assessed using the Shapiro–Wilk test. Between‐group differences at baseline were evaluated using Student's *t*‐tests or Wilcoxon rank‐sum tests, as appropriate. Linear mixed‐effects models (LMM) were used to evaluate intervention effects. LMM were estimated using restricted maximum likelihood and incorporated all available data without imputation, assuming data were missing at random. Model assumptions were evaluated by inspection of residuals to assess normality and homoscedasticity, which were deemed acceptable for all analyses. For circulating soluble RAGE and soluble TLR4, a 2 × 4 LMM (Group × Time) was applied across four timepoints: pre‐intervention baseline, pre‐intervention post‐acute exercise, post‐intervention baseline, and post‐intervention post‐acute exercise. For variables assessed only at baseline timepoints (pre—and post‐intervention), including body composition, glycemic markers, OGTT‐derived outcomes, insulin sensitivity indices, and skeletal muscle protein expression, a 2 × 2 LMM (Group × Time) was used. Model‐based estimated marginal means and planned contrasts were derived using the emmeans package and are presented as estimate ± SE. Planned contrasts were specified a priori to evaluate [1] acute exercise effects, [2] training effects, and [3] training‐induced changes. To maintain interpretability and align with study hypotheses, only biologically relevant, pre‐specified contrasts are reported in the main text, while full results for all contrasts are provided in the Supporting Information. Associations between changes in variables were assessed using Spearman correlation analyses, based on data distribution. These analyses were exploratory and not adjusted for multiple comparisons. Given the scope of the study and sample size, no formal adjustment for multiple comparisons across endpoints was performed; findings are interpreted accordingly. Statistical significance was set at *p* < 0.05, with results not meeting this threshold described descriptively and interpreted cautiously. Descriptive data are presented as mean ± SEM unless otherwise indicated. Standardized effect sizes for mixed‐model contrasts were calculated as the contrast estimate divided by the residual standard deviation from the fitted model. These model‐based effect sizes are distinct from Cohen's *d* values used for a priori power calculations, which were based on pooled standard deviations of change scores.

## 3 Results

### 3.1 Baseline Participant Characteristics

Baseline CON and AET group profiles are reported in Table 1. Participant race was 88% White, 8% Black, and 4% Asian, while ethnicity was 100% non‐Hispanic or Latino. There were no significant baseline differences between groups for demographic, anthropometric, cardiovascular, or metabolic characteristics. Medication use by group is summarized in Table S1. Participants were treated with a range of anti‐hyperglycemic medications, including metformin, insulin, GLP‐1 receptor agonists, SGLT2 inhibitors, and sulfonylureas, as well as common cardiovascular medications. Medication regimens remained generally stable throughout the intervention, with only one participant reporting a reduction in sulfonylurea dosage during the study period.

**TABLE 1:** Baseline subject characteristics.

Variable, units	CON	AET	*p*
*n*, % female	13, 69%	20, 70%	—
Age, year	59.6 ± 2.6	54.5 ± 2.1	0.135
Height, *m*	1.68 ± 0.03	1.67 ± 0.02	0.665
HbA1c, %	6.9 ± 0.2	7.5 ± 0.3	0.179
TSH, mIU/l (*W*)	1.9 ± 0.3	2.0 ± 0.4	0.703
Cholesterol, mg/dl	166 ± 11	172 ± 11	0.734
HDL, mg/dl (*W*)	45 ± 3	45 ± 3	0.683
LDL, mg/dl	88 ± 8	96 ± 9	0.484
Triglycerides, mg/dL (*W*)	168 ± 28	153 ± 19	0.849
Hb, g/dl	14.1 ± 0.4	13.7 ± 0.4	0.502
Hct, %	43 ± 1	42 ± 1	0.323

*Note:* Group differences assessed via two‐tailed *t*‐test or a Wilcoxon rank sum test (***W***). Data are presented as mean ± SEM.

Abbreviations: Hb: haemoglobin; Hct: haematocrit; HDL: high‐density lipoprotein; LDL: low‐density lipoprotein; TSH: thyroid stimulating hormone.

### 3.2 Anthropometrics

Baseline anthropometric characteristics were not different between groups for body weight, BMI, lean mass, fat mass, or body fat percentage (all *p* > 0.05; Table 2).

**TABLE 2:** Anthropometric, cardiovascular fitness, and glucose metabolism changes after the 12‐weeks intervention.

	CON baseline	CON post	AE baseline	AE post	Group (*p*)	Time (*p*)	Group×Time (*p*)	Cohen's *f*
Anthropometrics								
Weight, kg	91.8 ± 5.2	92.4 ± 5.2	96.4 ± 4.2	95.3 ± 4.3	0.580	0.674	0.235	0.248
BMI, kg/m^2^	32.6 ± 1.7	33.0 ± 1.7	34.6 ± 1.3	34.3 ± 1.4	0.453	0.992	0.148	0.319
Lean mass, kg	53.4 ± 3.0	52.8 ± 3.0	53.4 ± 2.4	54.4 ± 2.4	0.833	0.659	0.021*	0.575
Fat mass, kg	36.6 ± 3.5	36.9 ± 3.5	40.5 ± 2.8	39.0 ± 2.8^a^	0.503	0.110	0.014*	0.619
Body fat, %	40.7 ± 2.1	41.1 ± 2.1	42.7 ± 1.7	41.2 ± 1.7^a^	0.691	0.005*	*p* < 0.001*	1.22
Cardiovascular fitness								
VO_2_peak, mL/min	1929 ± 155	1933 ± 155	2184 ± 125	2289 ± 127	0.133	0.077	0.102	0.345
VO_2_peak, mL/kg/min	21.1 ± 1.3	21.4 ± 1.3	22.9 ± 1.0	24.7 ± 1.1^a^	0.127	0.005*	0.033*	0.460
Glucose metabolism								
Fasting glucose, mg/dL	143 ± 11	145 ± 11	158 ± 9	142 ± 10	0.647	0.303	0.163	0.302
Fasting insulin, uIU/mL	12.5 ± 2.8	12.0 ± 2.9	13.4 ± 2.4	11.1 ± 2.7	0.993	0.375	0.575	0.133
Fasting C‐peptide, pmol/L	767 ± 79	744 ± 81	825 ± 71	703 ± 79	0.933	0.189	0.367	0.194
Fasting NEFAs, umol/L	415 ± 63	477 ± 66	538 ± 54	386 ± 66	0.817	0.427	0.069	0.402
2‐h glucose, mg/dL	275 ± 20	267 ± 20	278 ± 16	255 ± 18	0.857	0.129	0.459	0.157
2‐h insulin, uIU/mL	36.0 ± 8.7	40.0 ± 8.9	40.0 ± 7.2	41.1 ± 8.3	0.811	0.608	0.768	0.063
2‐h C‐peptide, pmol/L	1763 ± 199	1897 ± 195	1790 ± 181	1779 ± 200	0.855	0.606	0.544	0.139
2‐h NEFAs, umol/L	128 ± 34	91 ± 33	149 ± 29	95 ± 35	0.752	0.081	0.732	0.073
Glucose niAUC, mg/dl* 120 min	13 797 ± 950	12 084 ± 979	12 947 ± 766	11 719 ± 906	0.587	0.030*	0.705	0.077
Insulin niAUC, uIU/mL* 120 min	2284 ± 751	3213 ± 766	2835 ± 621	2740 ± 704	0.966	0.309	0.215	0.258
C‐peptide niAUC, pmol/L* 120 min	88 410 ± 22 130	101 215 ± 22 130	76 199 ± 20 789	67 896 ± 22 166	0.437	0.840	0.351	0.240
NEFAs niAUC, umol/L* 120 min	−16 362 ± 5546	−25 394 ± 5546	−25 394 ± 4922	−16 807 ± 6201	0.750	0.080	0.730	0.398
HOMA‐IR (AU)	4.2 ± 1.1	4.3 ± 1.1	5.3 ± 0.9	4.1 ± 1.0	0.735	0.288	0.275	0.259
C‐peptidogenic index (AU)	4.8 ± 2.3	5.4 ± 2.3	6.9 ± 2.1	5.4 ± 2.2	0.732	0.585	0.204	0.295
Matsuda index (AU)	3.3 ± 0.8	3.3 ± 0.86	3.5 ± 0.7	6.2 ± 0.9^a^	0.093	0.070	0.055	0.373
Oral disposition index (AU)	13.6 ± 6.2	20.1 ± 6.4	20.3 ± 5.9	20.1 ± 6.4	0.582	0.303	0.565	0.127
Fructosamine, μM	431 ± 40	452 ± 40	489 ± 33	396 ± 38	0.984	0.158	0.026*	0.468

*Note:* Data are presented as model‐based estimated marginal means ± SE derived from linear mixed‐effects models. Group, time, and group × time *p* reflect fixed effects from the mixed‐effects model. Planned contrasts were obtained using the emmeans framework. Cohen's f was calculated to indicate effect size. No formal adjustment for multiple comparisons across endpoints was applied.

Abbreviations: BMI: body mass index; HOMA‐IR: homeostatic model assessment of insulin resistance; iAUC: incremental area under the curve; OGTT: oral glucose tolerance test; VO2peak: peak oxygen consumption.

* *p* < 0.05.

^a^ difference in AE post Versus pre. No other differences between CON or AE and CON observed.

There were significant group × time interactions for lean mass (*p* = 0.021), fat mass (*p* = 0.014), and body fat percentage (*p* < 0.001), indicating favourable body composition changes following AET relative to CON. Effect sizes for these interaction terms were moderate‐to‐large (Table 2). Consistent with these interaction effects, planned contrasts demonstrated that AET reduced fat mass by 1.52 ± 0.50 kg (*p* = 0.032) and body fat percentage by 1.46% ± 0.25% (*p* < 0.001). Lean mass increased numerically following AET by 0.93 ± 0.45 kg, although this contrast did not reach statistical significance (*p* = 0.208). No significant pre‐to‐post changes were observed in the CON group for anthropometric outcomes. No significant group × time interactions were observed for body weight (*p* = 0.235) or BMI (*p* = 0.148). A main effect of time was observed for body fat percentage (*p* = 0.005), reflecting an overall reduction across the study period.

### 3.3 Cardiovascular Outcomes

Baseline cardiovascular outcomes were not different between groups for relative VO_2_peak, absolute VO_2_peak, or peak heart rate achieved during exercise testing (all *p* > 0.05). There was a significant group × time interaction for relative VO_2_peak (*p* = 0.033), indicating improved cardiorespiratory fitness following AET relative to CON. The magnitude of this interaction was moderate (partial *η* ^2^ = 0.175; Cohen's *f* = 0.460). Planned contrasts demonstrated that AET increased relative VO_2_peak by 1.81 ± 0.46 mL·kg^−1^·min^−1^ (*p* = 0.003), whereas no significant change was observed in CON. A main effect of time was also observed for relative VO_2_peak (*p* = 0.005). No significant group × time interaction was observed for absolute VO_2_peak (*p* = 0.103), although moderate effect sizes were noted (Table 2). Planned contrasts demonstrated a numerical increase in absolute VO_2_peak following AET of 105 ± 40 mL/min that did not reach statistical significance (*p* = 0.068). There were no significant main effects of group or time for absolute VO_2_peak (all *p* > 0.05). Peak heart rate did not differ by group, time, or group × time interaction (all *p* > 0.05), and no significant pre‐to‐post changes were observed following AET or CON, supporting comparable maximal effort across testing timepoints.

### 3.4 Glucose Metabolism Outcomes

Baseline measures of glucose metabolism were not different between groups (all *p* > 0.05; Table 2). There was a significant group × time interaction for fructosamine (*p* = 0.026), indicating improved short‐term glycemic control following AET relative to CON. Planned contrasts demonstrated a reduction in fructosamine of 92.8 ± 34.1 μmol/L following AET that approached statistical significance (*p* = 0.051), whereas no significant change was observed in CON. A significant main effect of time was observed for glucose net iAUC (*p* = 0.030), reflecting overall reductions in glycemic excursion during the OGTT. No significant group × time interactions were observed for fasting plasma glucose (*p* = 0.163), fasting insulin (*p* = 0.575), HOMA‐IR (*p* = 0.275), Matsuda index (*p* = 0.055), c‐peptidogenic index (*p* = 0.204), oral disposition index (*p* = 0.565), insulin net iAUC (*p* = 0.215), C‐peptide net iAUC (*p* = 0.351), or NEFA net iAUC (*p* = 0.730). However, several outcomes demonstrated moderate effect sizes despite not reaching statistical significance, including Matsuda index (partial *η* ^2^ = 0.122; Cohen's *f* = 0.373) and fasting NEFA concentrations (partial *η* ^2^ = 0.139; Cohen's *f* = 0.402). Consistent with these effect sizes, planned contrasts demonstrated that AET increased Matsuda index by 2.75 ± 0.98 units (*p* = 0.043), suggesting improved whole‐body insulin sensitivity. Fasting glucose decreased numerically following AET by 16.0 ± 8.8 mg/dL, although this change was not statistically significant (*p* = 0.291). No significant pre‐to‐post changes were observed in the CON group for glucose metabolism outcomes.

### 3.5 Inflammatory Outcomes

**FIGURE 3:**
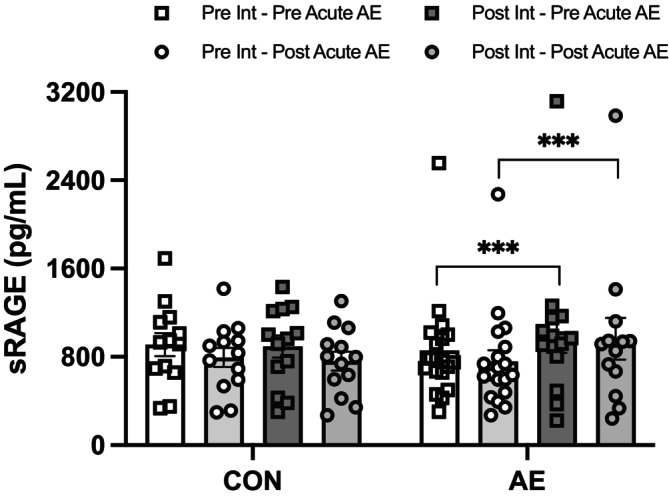
Solubilized receptor for advanced glycation end products (sRAGE; A), solubilized Toll‐like receptor 4 (sTLR4; B) pre and post‐12‐weeks intervention and acute aerobic exercise responses in the control (CON) and aerobic exercise training (AET) groups. Data were analysed using linear mixed‐effects models with fixed effects for group, time, and their interaction, with participant included as a random effect. Planned contrasts were specified a priori to evaluate acute exercise effects, intervention effects, and intervention‐induced changes in the acute exercise response.

Baseline circulating sRAGE and sTLR4 concentrations were not different between CON and AET (both *p* > 0.05). For sRAGE, there was a significant main effect of time (p < 0.001) and a significant group × time interaction (*p* < 0.001; Figure 3), indicating that circulating sRAGE changed differentially across the four sampling timepoints in AET Versus CON. Planned contrasts demonstrated that AET increased baseline sRAGE from pre—to post‐intervention by 213.0 ± 46.4 pg/mL (*p* < 0.001). Post intervention post‐acute exercise sRAGE was also higher following AET by 242.1 ± 46.4 pg/mL compared with the pre‐intervention post‐acute exercise timepoint (*p* < 0.001). Acute exercise reduced sRAGE before training in both CON and AET, as indicated by lower post‐exercise values relative to resting pre‐exercise values in CON (−115.3 ± 48.4 pg/mL, *p* = 0.020) and AET (−96.6 ± 40.1 pg/mL, *p* = 0.018). In CON, resting sRAGE did not change across the intervention period (*p* = 0.739). For sTLR4, there were no significant main effects of group or timepoint and no significant group × timepoint interaction (all *p* > 0.05). Planned contrasts similarly demonstrated no significant baseline group differences, acute exercise responses, or intervention‐related changes for sTLR4. Baseline hsCRP concentrations were not different between groups (*p* > 0.05). There were no significant group, time, or group × time effects for hsCRP (all *p* > 0.05). Consistent with this pattern, planned contrasts demonstrated a reduction in hsCRP following AET of 1.09 ± 0.45 mg/L, although this did not reach statistical significance (*p* = 0.096).

### 3.6 Muscle Protein Outcomes

**FIGURE 4:**
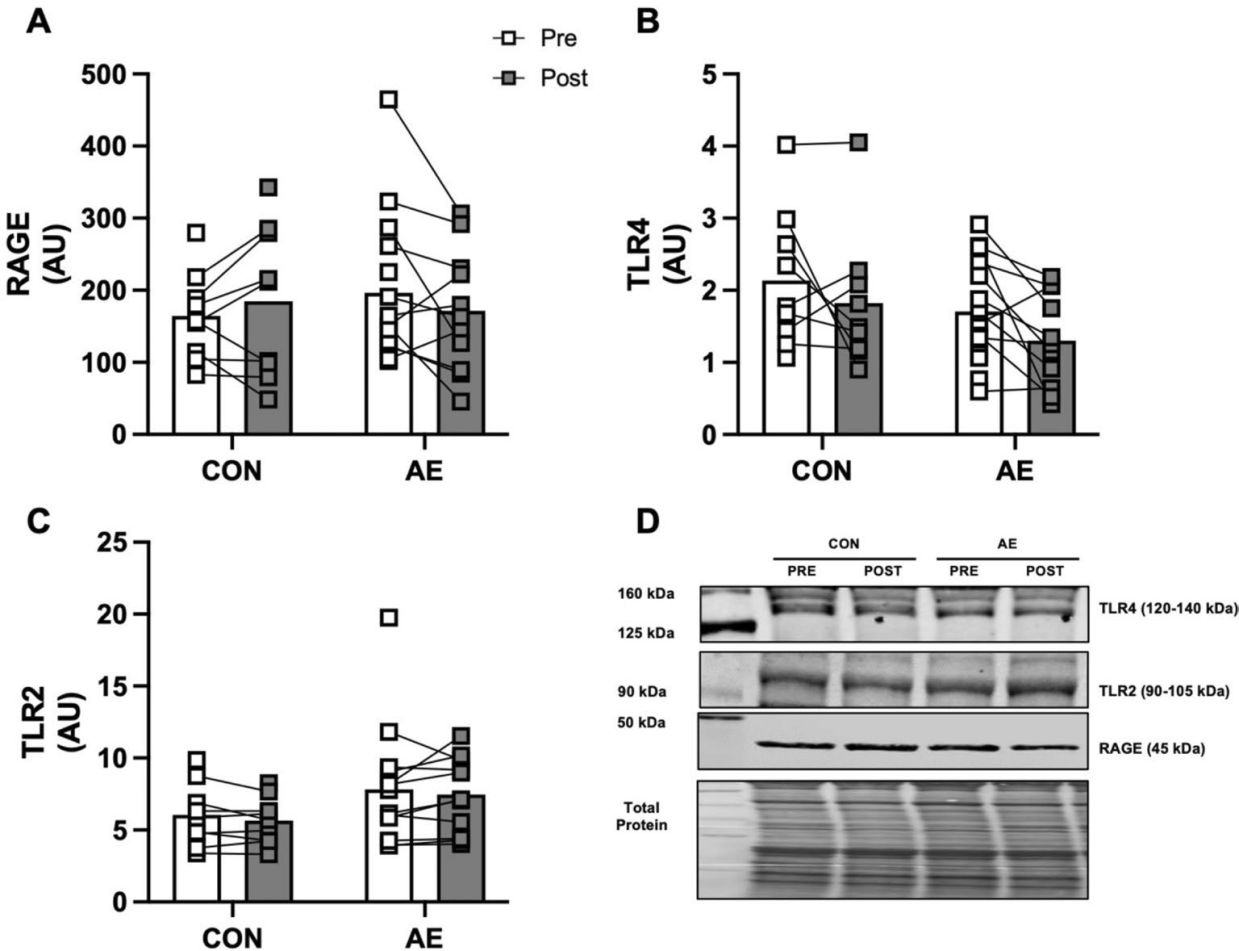
Vastus lateralis skeletal muscle receptor for advanced glycation end products (RAGE, A), Toll‐like receptor 4 (TLR4, B), and Toll‐like receptor 2 (TLR2, C) protein expression pre and post 12‐weeks intervention in the control (CON) and aerobic exercise training (AET) groups. Western‐blot quantification was normalized to total protein (D). Data were analysed using linear mixed‐effects models with fixed effects for group, time, and their interaction, with participant included as a random effect. Planned contrasts were specified a priori to evaluate intervention effects within and between groups.

**FIGURE 5:**
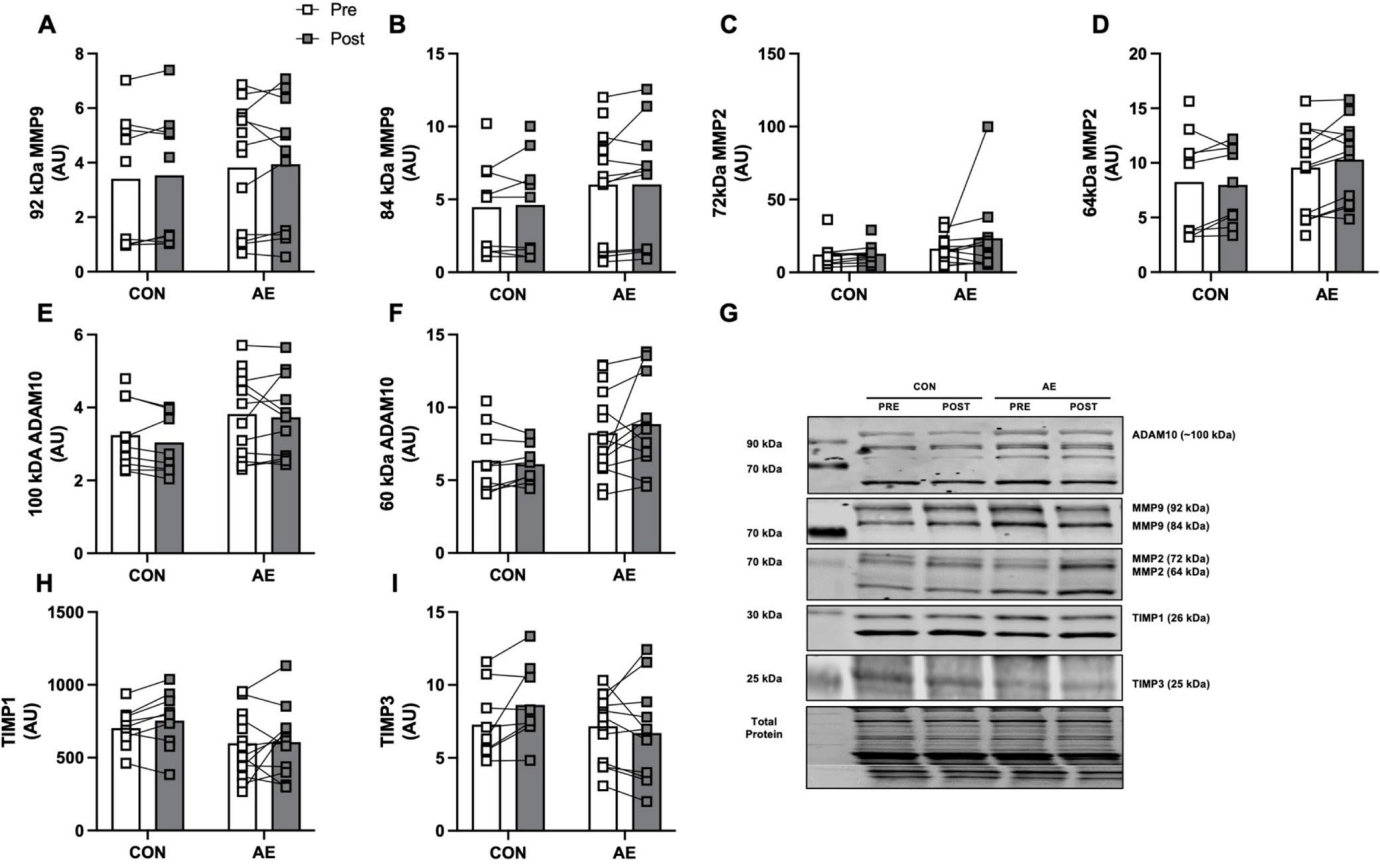
Basal vastus lateralis skeletal muscle pro (A) and active (B) matrix metalloproteinase (MMP) 9, pro (C) and active (D) MMP2, pro (E) and active (F) a disintegrin and metalloproteinase 10 (ADAM10), tissue inhibitors of metalloproteinases (TIMP) 1 (H) and TIMP3 (I) protein expression pre and post 12‐weeks intervention in the control (CON) and aerobic exercise training (AET) groups. Western‐blot quantification was normalized to total protein (G). Data were analysed using linear mixed‐effects models with fixed effects for group, time, and their interaction, with participant included as a random effect. Planned contrasts were specified a priori to evaluate intervention effects within and between groups.

Baseline skeletal muscle protein expression was not different between groups (*p* > 0.05). Among individual proteins, a significant main effect of time was observed for TLR4 expression (*p* = 0.028; Figure 4; partial *η* ^2^ = 0.224) and MMP2 64 kDa expression (*p* = 0.026; Figure 5; partial *η* ^2^ = 0.257). No significant group × time interactions were observed for individual proteins RAGE, TLR2, TLR4 (Figure 4), ADAM10, MMP2, MMP9, TIMP1, TIMP3 (Figure 5; all *p* > 0.05). For protein ratio outcomes, significant group × time interactions were observed for TIMP3:MMP9 84 kDa (*p* = 0.014; partial *η* ^2^ = 0.298) and TIMP3:ADAM10 100 kDa (*p* = 0.028; partial *η* ^2^ = 0.239). Significant group effects were also observed for TIMP1:ADAM10 60 kDa (*p* = 0.005), TIMP1:ADAM10 100 kDa (*p* = 0.012), TIMP3:MMP2 72 kDa (*p* = 0.010), TIMP3:ADAM10 60 kDa (*p* = 0.009), and TIMP3:ADAM10 100 kDa (*p* = 0.025). Planned contrasts demonstrated no significant pre‐to‐post changes following AET for individual protein expression outcomes. ADAM10 activity was unchanged. Basal ADAM10 activity was similar between groups at baseline (*p* > 0.05). No significant group × time interactions were observed for ADAM10 activity (*p* > 0.05).

### 3.7 Exploratory Correlation Analyses

Exploratory correlation analyses were performed to examine relationships between changes in circulating sRAGE and relevant metabolic, inflammatory, and exercise‐related outcomes to reduce redundancy and avoid overinterpretation of highly collinear variables; only relevant associations are highlighted herein. Among the observed associations, increases in circulating sRAGE were positively associated with changes in aerobic fitness (VO2peak; *r* = 0.451, *p* = 0.024) and C‐peptide iAUC (*r* = 0.515, *p* = 0.035), while inversely associated with circulating sTLR4 (*r* = −0.454, *p* = 0.020). Additional analyses demonstrated positive associations between membrane‐associated RAGE and TLR4 (*r* = 0.781, *p* < 0.001). A complete correlation matrix and heat map are provided in the Supporting Information for comprehensive interpretation (Figure S1).

## 4 Discussion

This study investigated the effects of 12 weeks of supervised AET on circulating sRAGE, sTLR4, and related skeletal muscle inflammatory signalling pathways in adults with T2DM. The primary finding was that AET increased circulating sRAGE concentrations by approximately 25%, with most participants demonstrating a positive training response. Exercise training also improved cardiorespiratory fitness, reduced body fat percentage, and improved select indices of glucose regulation, including fructosamine and the Matsuda Index. In contrast, acute exercise did not increase circulating sRAGE or sTLR4 at the sampled timepoint, and skeletal muscle protein responses were comparatively modest. Collectively, these findings suggest that AET may augment endogenous anti‐inflammatory defences in T2DM through mechanisms associated with soluble RAGE regulation, while also highlighting the complexity of tissue‐specific inflammatory adaptations in humans with metabolic disease.

sRAGE is increasingly recognized as a potentially protective circulating decoy receptor capable of binding AGEs and other inflammatory ligands, thereby limiting downstream RAGE signalling [57, 58]. Lower circulating sRAGE concentrations have consistently been associated with obesity, insulin resistance, vascular dysfunction, and progression toward T2DM [59, 60, 61, 62, 63, 64]. In the present study, AET increased circulating sRAGE by approximately 25%, which is among the larger intervention‐associated increases reported in adults with metabolic disease [39, 43, 44]. Importantly, this response occurred despite only modest changes in body weight, suggesting that exercise‐induced alterations in sRAGE may not simply reflect weight loss alone. In support of this concept, increases in sRAGE were associated with improvements in aerobic fitness, reinforcing the possibility that physiologic adaptation to exercise training itself may contribute to altered regulation of circulating sRAGE.

The current findings also help contextualize the mixed literature regarding exercise and sRAGE regulation. While several studies report exercise‐induced increases in circulating sRAGE [41, 65], others demonstrate reductions or no change depending on the population studied and exercise stimulus employed [39]. These discrepancies suggest that the sRAGE response to exercise is likely influenced by disease phenotype, baseline inflammatory burden, exercise programming, and timing of sample collection. Our cohort consisted exclusively of adults with established T2DM, many of whom were overweight or obese and treated with multiple anti‐hyperglycemic medications, representing a clinically complex population in which inflammatory signaling pathways may respond differently than in healthy adults or younger cohorts.

One notable finding was that acute exercise did not increase circulating sRAGE at the 30‐min post‐exercise sampling timepoint, either before or after the training intervention. In fact, modest reductions in sRAGE were observed immediately following the acute bout. While this contrasts with some prior reports of acute exercise‐induced elevations in sRAGE [41, 65], interpretation should be cautious given the limited temporal sampling design. Many inflammatory and receptor shedding‐related responses peak beyond 30–60 min following exercise, and it is plausible that the present study underdected the full kinetic response. Thus, the current data may suggest that the increase in resting sRAGE following training reflects a chronic adaptation to repeated exercise exposure rather than an immediate transient response detectable shortly after a single bout of exercise.

We originally hypothesized that exercise‐induced increases in sRAGE may be associated with altered sheddase regulation involving ADAM10, MMPs, and TIMPs. While the current findings provide some support for altered regulation of these enzymes, the data do not establish a direct mechanistic link. Specifically, the marked reduction in the TIMP3:ADAM10 ratio following AET may reflect a shift toward a less inhibitory sheddase environment; however, ADAM10 activity itself did not significantly increase. Similarly, while changes in circulating sRAGE were associated with MMP9‐related outcomes, these findings remain associative and should be interpreted as hypothesis‐generating rather than mechanistic evidence of receptor shedding. Collectively, the data are consistent with the possibility that exercise training alters the regulatory milieu surrounding RAGE processing, but additional mechanistic studies with direct measures of shedding flux and tissue‐specific receptor turnover are needed.

Contrary to our hypothesis, skeletal muscle may not be the dominant contributor to circulating sRAGE adaptations following AET in this cohort. Although skeletal muscle was biologically relevant to study given its tissue mass, metabolic activity, and responsiveness to exercise, muscle RAGE protein expression itself did not significantly change following training. Likewise, many skeletal muscle inflammatory markers demonstrated relatively small effect sizes or substantial variability. Importantly, post hoc power analyses indicated that several muscle protein outcomes would require substantially larger sample sizes to confidently detect moderate intervention effects, whereas the study was appropriately powered for the primary circulating sRAGE outcome. For example, observed power estimates suggested that approximately 24 participants per group would be required to adequately detect changes in skeletal muscle RAGE protein expression. Thus, while the current data do not strongly support skeletal muscle as the exclusive source of exercise‐induced increases in circulating sRAGE, they also do not definitively exclude a contribution from skeletal muscle. Rather, the findings support the likelihood that multiple tissues, including adipose tissue, vascular endothelium, immune cells, and potentially liver, may contribute to circulating sRAGE regulation in response to exercise training [22, 41].

In contrast to sRAGE, circulating sTLR4 was not altered by aerobic exercise training. This finding was somewhat unexpected given prior reports demonstrating altered TLR4 signalling with exercise and metabolic dysfunction [65]. However, circulating soluble receptors may not necessarily parallel tissue‐specific receptor expression or signalling activity. Indeed, membrane‐associated TLR4 in skeletal muscle demonstrated a significant main effect of time, although no clear intervention‐specific effect emerged. Together, these findings may suggest that TLR4 regulation in T2DM is less responsive to AET than the RAGE axis, or alternatively, that soluble TLR4 regulation follows different temporal or tissue‐specific dynamics not captured in the present study.

The observed metabolic adaptations provide additional context for the inflammatory findings. AET improved cardiorespiratory fitness and reduced adiposity while also improving the Matsuda Index and fructosamine concentrations. Interestingly, improvements were more apparent in indices reflecting whole‐body or peripheral insulin sensitivity than fasting‐derived indices such as HOMA‐IR. This pattern is physiologically plausible given that exercise training preferentially enhances skeletal muscle glucose disposal and peripheral insulin action [66, 67, 68]. Although the study was not designed to mechanistically link sRAGE changes to improvements in glucose metabolism, the associations between aerobic fitness and circulating sRAGE raise the possibility that exercise‐induced improvements in metabolic health and inflammatory regulation may occur in parallel.

Several considerations should be acknowledged when interpreting these findings. First, the study was powered a priori for the primary circulating sRAGE outcome rather than the secondary skeletal muscle protein endpoints, which were comparatively variable and may have been underpowered to detect modest biologically relevant effects. Second, acute exercise responses were assessed at a single post‐exercise timepoint and therefore may not have fully captured the temporal dynamics of inflammatory signalling or receptor shedding following exercise. Third, although skeletal muscle biopsies provided valuable tissue‐level insight into exercise‐responsive inflammatory pathways, circulating sRAGE likely reflects contributions from multiple tissues and organ systems beyond skeletal muscle alone, which could not be determined in the present study. In addition, dietary intake and non‐exercise physical activity were not strictly controlled and participants were also treated with a range of anti‐hyperglycemic and cardiovascular medications, which may have influenced inflammatory and metabolic responses despite relative stability of medication use throughout the intervention. The cohort was also predominantly White and non‐Hispanic, and the intervention consisted of intensive supervised aerobic exercise, which may limit broader generalizability. Finally, the exploratory correlation analyses presented herein are associative in nature and should not be interpreted as evidence of causality or direct mechanistic regulation.

In conclusion, AET increased circulating sRAGE concentrations and improved several markers of metabolic health in adults with T2DM. These findings are consistent with the concept that chronic exercise training may favourably influence endogenous anti‐inflammatory defence systems involving the RAGE axis. However, the underlying mechanisms responsible for exercise‐induced increases in circulating sRAGE remain incompletely understood. The present findings support further mechanistic investigation into tissue‐specific regulation of soluble RAGE and its potential role as a biomarker or mediator of exercise responsiveness in metabolic disease.

## Funding

This research was supported by the National Institutes of Health, National Institute of Diabetes and Digestive and Kidney Diseases (NIDDK) under award R01 DK109948 (J.M.H.). Core services used in this study were supported by NIDDK grants P30 DK020572 (Michigan Diabetes Research Center) and P30 DK089503 (Michigan Nutrition Obesity Research Center) awarded to the University of Michigan.

## Conflicts of Interest

The authors declare no conflicts of interest.

## Supporting information


**Table S1:** Medication usage by group.
**Table S2:** Primary and secondary antibodies.
**Figure S1:** Correlation heat map of baseline metabolic, inflammatory, and skeletal muscle signalling variables. Spearman correlation coefficients (*r*) are displayed for pairwise associations among anthropometric, metabolic, inflammatory, circulating receptor, and skeletal muscle protein outcomes. Colour intensity represents the magnitude and direction of the correlation, with red indicating positive correlations and blue indicating negative correlations. Only the lower triangle of the correlation matrix is displayed to improve visualization. Correlation coefficients are shown within cells where statistically significant associations were identified (*p* < 0.05). Corresponding correlation coefficients and *p* values for all pairwise comparisons are provided in Supporting Information.

## Data Availability

Data available on request from the authors The data that support the findings of this study are available from the corresponding author upon reasonable request.
