# Supplementary material for: Aerobic Exercise Training Increases Circulating sRAGE in Adults With Type 2 Diabetes: Associations With Sheddase Regulation
Source: Diabetes Obes Metab. 2026 Jun 17;28(9):8157–71. doi: 10.1111/dom.70995 (PMC13448878; doi:10.1111/dom.70995)
Supplement: Supplementary file 1 [file DOM-28-8157-s001.pdf]

Supplemental Table 1. Medication usage by group.

Medication Class	CON (n = 13)	AET (n = 20)	Total (n = 33)
Glucose-lowering medications			
Metformin	10 (77%)	18 (85%)	28 (88%)
Insulin	2 (15%)	2 (11%)	5 (13%)
GLP-1 receptor agonists	1 (8%)	2 (5%)	3 (9%)
SGLT2 inhibitors	2 (15%)	2 (11%)	4 (13%)
Sulfonylureas	1 (8%)	3 (16%)	4 (13%)
DPP-4 inhibitors	0 (0%)	3 (16%)	3 (9%)
Cardiovascular medications			
Beta-blockers	1 (8%)	2 (11%)	3 (9%)
ACE inhibitors / ARBs	6 (46%)	8 (42%)	14 (44%)
Calcium channel blockers	1 (8%)	1 (5%)	2 (6%)
Diuretics	4 (31%)	3 (16%)	7 (22%)
Statins	7 (46%)	8 (42%)	15 (47%)
Aspirin	3 (23%)	2 (11%)	5 (16%)
Other medications			
Antidepressants (SSRI/SNRI)	2 (15%)	4 (21%)	6 (19%)
Thyroid medications	1 (8%)	2 (11%)	3 (9%)
Bronchodilators	1 (8%)	0 (0%)	1 (3%)

Values are presented as n (% of group). Participants may be taking multiple medications. Medication classifications were assigned based on reported prescription details and include both generic and brand names. ACE, angiotensin-converting enzyme; ARB, angiotensin II receptor blocker; GLP-1, glucagon-like peptide-1; SGLT2, sodium–glucose cotransporter 2; DPP-4, dipeptidyl peptidase-4.

Supplemental Table 2. Primary and secondary antibodies.

Antibody	Company	Catalog Number	Dilution
RAGE	Abcam	ab3611	1:1000
ADAM10	CST	14194	1:1000
TLR2	CST	12276	1:1000
TLR4	CST	38519	1:1250
MMP2	CST	40994	1:1000
MMP9	CST	13667	1:1000
TIMP 1	CST	8946	1:1000
TIMP 3	CST	5673	1:500
IRDye 800CW Donkey anti-Mouse IgG Secondary Antibody	LI-COR	926-32212	1:10000
IRDye 800CW Donkey anti-Rabbit IgG Secondary Antibody	LI-COR	926-32213	1:10000

Receptor for advanced glycation end products, RAGE; a disintegrin and metalloproteinase 10, ADAM10; Toll-like receptor, TLR; matrix metalloproteinase, MMP; tissue inhibitors of metalloproteinases (TIMPs).

Supplemental Figure 1

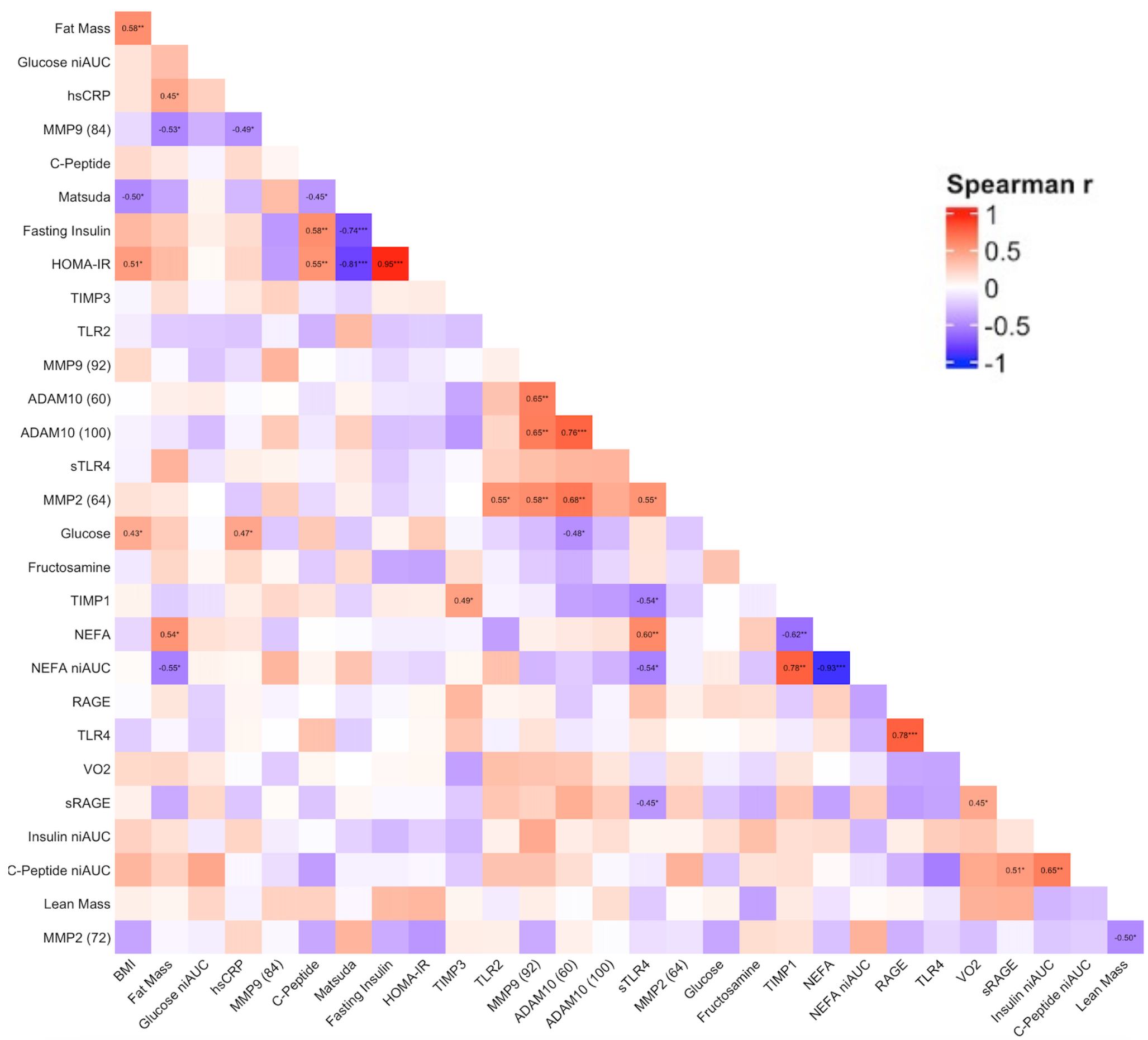

Outcome	contrast	p.value	estimate	SE	df	t.ratio	Model_Sigma	Std_Estimate_ModelSigma
Weight (kg)	CON Pre - AE Pre	0.904414	-4.56473	6.726268	31.4009	-0.67864	2.430117241	-1.8783994
Weight (kg)	CON Pre - CON Post	0.943089	-0.53308	0.95317	24.00037	-0.55927	2.430117241	-0.219362636
Weight (kg)	CON Pre - AE Post	0.955537	-3.45834	6.749726	31.83077	-0.51237	2.430117241	-1.423115524
Weight (kg)	AE Pre - CON Post	0.931479	4.031654	6.726268	31.4009	0.599389	2.430117241	1.659036765
Weight (kg)	AE Pre - AE Post	0.6558	1.106393	0.952191	24.13949	1.161944	2.430117241	0.455283876
Weight (kg)	CON Post - AE Post	0.972276	-2.92526	6.749726	31.83077	-0.43339	2.430117241	-1.203752889
BMI	CON Pre - AE Pre	0.801561	-1.9264	2.12542	31.32902	-0.90636	0.726886787	-2.650199655
BMI	CON Pre - CON Post	0.720502	-0.31254	0.296692	22.02537	-1.05341	0.726886787	-0.429967977
BMI	CON Pre - AE Post	0.874119	-1.60956	2.133648	31.8058	-0.75437	0.726886787	-2.214315376
BMI	AE Pre - CON Post	0.872231	1.613857	2.127005	31.42085	0.758746	0.726886787	2.220231678
BMI	AE Pre - AE Post	0.711548	0.316839	0.296445	22.13113	1.068793	0.726886787	0.435884279
BMI	CON Post - AE Post	0.928966	-1.29702	2.135227	31.89728	-0.60744	0.726886787	-1.784347399
Lean Mass (kg)	CON Pre - AE Pre	1	-0.02917	3.834721	31.19173	-0.00761	1.014638306	-0.028753633
Lean Mass (kg)	CON Pre - CON Post	0.461666	0.645147	0.432545	19.02611	1.491514	1.014638306	0.635839607
Lean Mass (kg)	CON Pre - AE Post	0.994514	-0.95471	3.845408	31.53505	-0.24827	1.014638306	-0.940933851
Lean Mass (kg)	AE Pre - CON Post	0.998032	0.674322	3.838463	31.31211	0.175675	1.014638306	0.66459324
Lean Mass (kg)	AE Pre - AE Post	0.207909	-0.92553	0.453306	19.115	-2.04174	1.014638306	-0.912180218
Lean Mass (kg)	CON Post - AE Post	0.975394	-1.59985	3.849139	31.65496	-0.41564	1.014638306	-1.576773459
Fat Mass (kg)	CON Pre - AE Pre	0.811651	-3.96364	4.469329	31.17424	-0.88685	1.127496731	-3.515437016
Fat Mass (kg)	CON Pre - CON Post	0.878667	-0.35692	0.480667	19.02372	-0.74255	1.127496731	-0.316559356
Fat Mass (kg)	CON Pre - AE Post	0.947357	-2.4395	4.480656	31.48667	-0.54445	1.127496731	-2.163640813
Fat Mass (kg)	AE Pre - CON Post	0.85098	3.606724	4.473295	31.28376	0.806279	1.127496731	3.19887766
Fat Mass (kg)	AE Pre - AE Post	0.03216	1.524146	0.503773	19.10453	3.025461	1.127496731	1.351796204
Fat Mass (kg)	CON Post - AE Post	0.966267	-2.08258	4.484612	31.5958	-0.46438	1.127496731	-1.847081457
Body Fat (%)	CON Pre - AE Pre	0.881636	-2.0078	2.726183	31.11343	-0.73649	0.5551799	-3.616494173
Body Fat (%)	CON Pre - CON Post	0.432627	-0.36536	0.236698	19.01543	-1.54357	0.5551799	-0.658091996
Body Fat (%)	CON Pre - AE Post	0.997147	-0.54355	2.730693	31.31776	-0.19905	0.5551799	-0.979047274
Body Fat (%)	AE Pre - CON Post	0.930622	1.642445	2.727762	31.18499	0.602122	0.5551799	2.958402177
Body Fat (%)	AE Pre - AE Post	5.99E-05	1.464258	0.248137	19.06808	5.901006	0.5551799	2.637446899
Body Fat (%)	CON Post - AE Post	0.999898	-0.17819	2.732268	31.38916	-0.06522	0.5551799	-0.320955278
VO2 Peak Relative (mL/kg	CON Pre - AE Pre	0.703587	-1.78231	1.649318	32.72521	-1.08063	1.220263328	-1.460592686
VO2 Peak Relative (mL/kg	CON Pre - CON Post	0.943447	-0.27771	0.497691	24.13214	-0.558	1.220263328	-0.227583397
VO2 Peak Relative (mL/kg	CON Pre - AE Post	0.156778	-3.59347	1.668074	34.0909	-2.15427	1.220263328	-2.944834585
VO2 Peak Relative (mL/kg	AE Pre - CON Post	0.800093	1.504596	1.654951	33.13588	0.909148	1.220263328	1.233009289
VO2 Peak Relative (mL/kg	AE Pre - AE Post	0.003109	-1.81117	0.459482	24.5041	-3.94176	1.220263328	-1.484241899
VO2 Peak Relative (mL/kg	CON Post - AE Post	0.214707	-3.31576	1.673643	34.49517	-1.98116	1.220263328	-2.717251188
VO2 Peak Absolute (mL/m	CON Pre - AE Pre	0.581129	-255.199	199.1563	31.89015	-1.2814	106.6922279	-2.391920317
VO2 Peak Absolute (mL/m	CON Pre - CON Post	0.999616	-4.41276	43.53517	24.06831	-0.10136	106.6922279	-0.041359734
VO2 Peak Absolute (mL/m	CON Pre - AE Post	0.292856	-360.118	200.3616	32.62968	-1.79734	106.6922279	-3.375298539
VO2 Peak Absolute (mL/m	AE Pre - CON Post	0.596171	250.7865	199.5176	32.11183	1.256964	106.6922279	2.350560583
VO2 Peak Absolute (mL/m	AE Pre - AE Post	0.068482	-104.919	40.24728	24.26337	-2.60685	106.6922279	-0.983378222
VO2 Peak Absolute (mL/m	CON Post - AE Post	0.304542	-355.705	200.7207	32.85002	-1.77214	106.6922279	-3.333938805
HR Peak (bpm)	CON Pre - AE Pre	1	-0.02308	5.004253	36.67212	-0.00461	6.627269545	-0.003482116
HR Peak (bpm)	CON Pre - CON Post	0.897204	1.957037	2.807725	23.82488	0.697019	6.627269545	0.295300609

HR Peak (bpm)	CON Pre - AE Post	0.998365	0.855347	5.173798	40.1848	0.165323	6.627269545	0.129064719
HR Peak (bpm)	AE Pre - CON Post	0.980008	1.980114	5.115549	39.00713	0.387077	6.627269545	0.298782725
HR Peak (bpm)	AE Pre - AE Post	0.984276	0.878424	2.473399	24.58531	0.355148	6.627269545	0.132546835
HR Peak (bpm)	CON Post - AE Post	0.99674	-1.10169	5.281522	42.26462	-0.20859	6.627269545	-0.16623589
FPG (mg/dL)	CON Pre - AE Pre	0.695567	-15.2115	13.91275	40.28985	-1.09335	23.05022158	-0.659930249
FPG (mg/dL)	CON Pre - CON Post	0.993342	-2.47639	9.361109	23.74471	-0.26454	23.05022158	-0.107434455
FPG (mg/dL)	CON Pre - AE Post	0.99995	0.764389	14.77506	45.42895	0.051735	23.05022158	0.033161911
FPG (mg/dL)	AE Pre - CON Post	0.803947	12.73515	14.12284	41.64546	0.901742	23.05022158	0.552495794
FPG (mg/dL)	AE Pre - AE Post	0.291358	15.97593	8.824342	25.94988	1.810438	23.05022158	0.69309216
FPG (mg/dL)	CON Post - AE Post	0.996368	3.240777	14.97305	46.43766	0.216441	23.05022158	0.140596366
Fasting Insulin (0 min) (m	CON Pre - AE Pre	0.995639	-0.84629	3.680379	36.94205	-0.22995	5.39798185	-0.156778491
Fasting Insulin (0 min) (m	CON Pre - CON Post	0.995241	0.51784	2.194834	22.55382	0.235936	5.39798185	0.095932196
Fasting Insulin (0 min) (m	CON Pre - AE Post	0.983058	1.423099	3.891945	42.11738	0.365652	5.39798185	0.263635329
Fasting Insulin (0 min) (m	AE Pre - CON Post	0.982966	1.364128	3.725545	38.11875	0.366155	5.39798185	0.252710687
Fasting Insulin (0 min) (m	AE Pre - AE Post	0.722094	2.269386	2.160839	24.37225	1.050234	5.39798185	0.42041382
Fasting Insulin (0 min) (m	CON Post - AE Post	0.995643	0.905258	3.934683	43.04459	0.230072	5.39798185	0.167703133
Insulin 2-hour (120 min)	CON Pre - AE Pre	0.984091	-4.05084	11.32787	37.77189	-0.3576	17.48580635	-0.231664513
Insulin 2-hour (120 min)	CON Pre - CON Post	0.939631	-4.0594	7.106366	22.62395	-0.57123	17.48580635	-0.232154043
Insulin 2-hour (120 min)	CON Pre - AE Post	0.973387	-5.15038	12.0374	43.11285	-0.42786	17.48580635	-0.294546334
Insulin 2-hour (120 min)	AE Pre - CON Post	1	-0.00856	11.47964	39.00455	-0.00075	17.48580635	-0.00048953
Insulin 2-hour (120 min)	AE Pre - AE Post	0.998571	-1.09954	6.983057	24.6382	-0.15746	17.48580635	-0.06288182
Insulin 2-hour (120 min)	CON Post - AE Post	0.999738	-1.09098	12.18033	44.04407	-0.08957	17.48580635	-0.062392291
NEFA fasting (0 min) (uM)	CON Pre - AE Pre	0.462627	-122.184	83.07401	48.86051	-1.47078	200.7897777	-0.608515842
NEFA fasting (0 min) (uM)	CON Pre - CON Post	0.872768	-61.2504	80.91077	23.87745	-0.75701	200.7897777	-0.305047246
NEFA fasting (0 min) (uM)	CON Pre - AE Post	0.988824	29.05489	91.60785	50.16829	0.317166	200.7897777	0.144703034
NEFA fasting (0 min) (uM)	AE Pre - CON Post	0.890328	60.93339	85.11933	49.2597	0.715858	200.7897777	0.303468597
NEFA fasting (0 min) (uM)	AE Pre - AE Post	0.228224	151.2387	77.26786	27.86039	1.957329	200.7897777	0.753218876
NEFA fasting (0 min) (uM)	CON Post - AE Post	0.769133	90.30526	93.46662	50.34758	0.966177	200.7897777	0.44975028
HOMA-IR	CON Pre - AE Pre	0.882036	-1.03595	1.408075	35.0476	-0.73572	1.785507479	-0.580201325
HOMA-IR	CON Pre - CON Post	0.999996	-0.01627	0.726784	22.39768	-0.02239	1.785507479	-0.009112946
HOMA-IR	CON Pre - AE Post	0.999905	0.093996	1.470873	39.52178	0.063905	1.785507479	0.052643739
HOMA-IR	AE Pre - CON Post	0.88954	1.019683	1.421416	36.03295	0.717371	1.785507479	0.57108838
HOMA-IR	AE Pre - AE Post	0.412646	1.12995	0.718594	23.75466	1.572446	1.785507479	0.632845064
HOMA-IR	CON Post - AE Post	0.99985	0.110267	1.48365	40.37115	0.074321	1.785507479	0.061756685
Matsuda Index	CON Pre - AE Pre	0.999242	-0.13719	1.072633	49.18714	-0.1279	2.561232139	-0.053563891
Matsuda Index	CON Pre - CON Post	0.999808	0.083096	1.032813	23.88264	0.080456	2.561232139	0.032443948
Matsuda Index	CON Pre - AE Post	0.08695	-2.8839	1.194097	51.04325	-2.41513	2.561232139	-1.125979865
Matsuda Index	AE Pre - CON Post	0.997111	0.220286	1.099103	49.71823	0.200424	2.561232139	0.086007839
Matsuda Index	AE Pre - AE Post	0.043065	-2.74671	0.982773	28.44242	-2.79485	2.561232139	-1.072415975
Matsuda Index	CON Post - AE Post	0.082956	-2.96699	1.21793	51.26109	-2.43609	2.561232139	-1.158423814
Insulinogenic Index	CON Pre - AE Pre	0.853607	-0.14336	0.179002	42.43384	-0.80089	0.341998633	-0.4191842
Insulinogenic Index	CON Pre - CON Post	0.216265	-0.27749	0.138605	23.04743	-2.00204	0.341998633	-0.811384338
Insulinogenic Index	CON Pre - AE Post	0.823882	-0.1679	0.194604	47.41938	-0.86278	0.341998633	-0.490936969
Insulinogenic Index	AE Pre - CON Post	0.882261	-0.13413	0.182369	43.69127	-0.7355	0.341998633	-0.392200139
Insulinogenic Index	AE Pre - AE Post	0.997797	-0.02454	0.134688	26.10982	-0.18219	0.341998633	-0.07175277

Insulinogenic Index	CON Post - AE Post	0.944926	0.109593	0.197706	48.15854	0.554322	0.341998633	0.320447369
Disposition Index	CON Pre - AE Pre	0.857075	-0.34451	0.434208	47.631	-0.79342	0.985940264	-0.34942156
Disposition Index	CON Pre - CON Post	0.181973	-0.83632	0.398134	23.64182	-2.10059	0.985940264	-0.848243712
Disposition Index	CON Pre - AE Post	0.504814	-0.67387	0.481095	50.39945	-1.40071	0.985940264	-0.683483971
Disposition Index	AE Pre - CON Post	0.687201	-0.49181	0.444406	48.40124	-1.10667	0.985940264	-0.498822153
Disposition Index	AE Pre - AE Post	0.82306	-0.32937	0.381103	27.83916	-0.86424	0.985940264	-0.334062412
Disposition Index	CON Post - AE Post	0.987309	0.162443	0.490319	50.74184	0.331301	0.985940264	0.164759741
Glucose net iAUC	CON Pre - AE Pre	0.89752	851.1923	1220.513	43.38295	0.697405	2303.816269	0.369470569
Glucose net iAUC	CON Pre - CON Post	0.282131	1713.903	933.9581	24.021	1.835096	2303.816269	0.743940916
Glucose net iAUC	CON Pre - AE Post	0.397905	2077.891	1312.902	48.26973	1.58267	2303.816269	0.901934554
Glucose net iAUC	AE Pre - CON Post	0.898856	862.7109	1243.134	44.73661	0.693981	2303.816269	0.374470347
Glucose net iAUC	AE Pre - AE Post	0.508641	1226.699	874.5381	26.86048	1.402682	2303.816269	0.532463985
Glucose net iAUC	CON Post - AE Post	0.992808	363.9883	1333.957	49.14384	0.272864	2303.816269	0.157993638
Insulin net iAUC	CON Pre - AE Pre	0.941496	-551.825	974.8221	36.77551	-0.56608	1414.09879	-0.390230549
Insulin net iAUC	CON Pre - CON Post	0.389981	-929.398	575.0315	22.53989	-1.61626	1414.09879	-0.657236956
Insulin net iAUC	CON Pre - AE Post	0.970472	-456.935	1029.819	41.90772	-0.4437	1414.09879	-0.323127775
Insulin net iAUC	AE Pre - CON Post	0.980643	-377.573	986.5582	37.93898	-0.38272	1414.09879	-0.267006406
Insulin net iAUC	AE Pre - AE Post	0.99828	94.88995	566.3394	24.31858	0.16755	1414.09879	0.067102775
Insulin net iAUC	CON Post - AE Post	0.968514	472.4634	1040.935	42.83213	0.453884	1414.09879	0.334109181
NEFA net iAUC	CON Pre - AE Pre	0.619283	9031.894	7415.622	40.19782	1.217955	16197.82629	0.557599164
NEFA net iAUC	CON Pre - CON Post	0.582861	9031.521	7022.376	18.14278	1.286106	16197.82629	0.55757609
NEFA net iAUC	CON Pre - AE Post	0.999944	444.6976	8319.551	40.8355	0.053452	16197.82629	0.027454156
NEFA net iAUC	AE Pre - CON Post	1	-0.37374	7415.622	40.19782	-5E-05	16197.82629	-2.30735E-05
NEFA net iAUC	AE Pre - AE Post	0.656276	-8587.2	7398.323	25.65013	-1.16069	16197.82629	-0.530145008
NEFA net iAUC	CON Post - AE Post	0.731746	-8586.82	8319.551	40.8355	-1.03213	16197.82629	-0.530121934
C-peptide net iAUC	CON Pre - AE Pre	0.977576	12210.05	30362.51	28.76474	0.402142	32428.13694	0.3765263
C-peptide net iAUC	CON Pre - CON Post	0.811044	-12805.3	14418.95	15.58553	-0.88809	32428.13694	-0.394882527
C-peptide net iAUC	CON Pre - AE Post	0.91301	20514.04	31321.95	31.10689	0.654941	32428.13694	0.632599904
C-peptide net iAUC	AE Pre - CON Post	0.84269	-25015.4	30362.51	28.76474	-0.82389	32428.13694	-0.771408827
C-peptide net iAUC	AE Pre - AE Post	0.959821	8303.99	16874.85	17.47182	0.492093	32428.13694	0.256073604
C-peptide net iAUC	CON Post - AE Post	0.71366	33319.34	31321.95	31.10689	1.06377	32428.13694	1.027482431
Fructosamine	CON Pre - AE Pre	0.677954	-58.3394	51.9766	41.49442	-1.12242	89.25530958	-0.653623362
Fructosamine	CON Pre - CON Post	0.922817	-21.9035	35.00881	24.20554	-0.62566	89.25530958	-0.245402994
Fructosamine	CON Pre - AE Post	0.924292	34.48531	55.38139	46.7177	0.622688	89.25530958	0.386367083
Fructosamine	AE Pre - CON Post	0.896097	36.43584	51.9766	41.49442	0.701005	89.25530958	0.408220368
Fructosamine	AE Pre - AE Post	0.051334	92.82467	34.0908	27.25688	2.722866	89.25530958	1.039990446
Fructosamine	CON Post - AE Post	0.739682	56.38883	55.38139	46.7177	1.018191	89.25530958	0.631770077
TIMP1	CON Pre - AE Pre	0.717115	90.30149	85.34675	29.59663	1.058054	108.7824055	0.830111191
TIMP1	CON Pre - CON Post	0.732687	-55.5222	53.74503	18.15896	-1.03307	108.7824055	-0.51039654
TIMP1	CON Pre - AE Post	0.941434	49.996	88.33708	31.99373	0.565969	108.7824055	0.459596424
TIMP1	AE Pre - CON Post	0.337306	-145.824	85.34675	29.59663	-1.7086	108.7824055	-1.340507731
TIMP1	AE Pre - AE Post	0.815051	-40.3055	45.79506	18.32106	-0.88013	108.7824055	-0.370514766
TIMP1	CON Post - AE Post	0.63469	105.5182	88.33708	31.99373	1.194495	108.7824055	0.969992965
TIMP3	CON Pre - AE Pre	0.999977	0.041911	1.059139	28.75575	0.039571	1.262838006	0.033188157
TIMP3	CON Pre - CON Post	0.210505	-1.27498	0.62498	18.0142	-2.04003	1.262838006	-1.009612408

TIMP3	CON Pre - AE Post	0.990845	0.322713	1.09216	31.0237	0.295481	1.262838006	0.255545755
TIMP3	AE Pre - CON Post	0.605183	-1.31689	1.059139	28.75575	-1.24336	1.262838006	-1.042800565
TIMP3	AE Pre - AE Post	0.951373	0.280802	0.53261	18.14684	0.527218	1.262838006	0.222357598
TIMP3	CON Post - AE Post	0.471344	1.59769	1.09216	31.0237	1.462871	1.262838006	1.265158163
MMP9 92kDa	CON Pre - AE Pre	0.980573	-0.34916	0.913981	23.75452	-0.38202	0.402311051	-0.867894192
MMP9 92kDa	CON Pre - CON Post	0.920412	-0.1269	0.20091	17.13747	-0.63164	0.402311051	-0.315433015
MMP9 92kDa	CON Pre - AE Post	0.95865	-0.45768	0.918222	24.17548	-0.49844	0.402311051	-1.137614835
MMP9 92kDa	AE Pre - CON Post	0.994805	0.222261	0.913981	23.75452	0.243179	0.402311051	0.552461178
MMP9 92kDa	AE Pre - AE Post	0.919828	-0.10851	0.171323	17.14782	-0.63337	0.402311051	-0.269720642
MMP9 92kDa	CON Post - AE Post	0.983609	-0.33077	0.918222	24.17548	-0.36023	0.402311051	-0.82218182
MMP9 84kDa	CON Pre - AE Pre	0.910718	-1.02858	1.556873	23.66109	-0.66067	0.641802819	-1.602648854
MMP9 84kDa	CON Pre - CON Post	0.933508	-0.18938	0.320559	17.12055	-0.59077	0.641802819	-0.295071766
MMP9 84kDa	CON Pre - AE Post	0.764643	-1.52448	1.563219	24.03161	-0.97522	0.641802819	-2.375308749
MMP9 84kDa	AE Pre - CON Post	0.948571	0.839207	1.556873	23.66109	0.539034	0.641802819	1.307577088
MMP9 84kDa	AE Pre - AE Post	0.300695	-0.4959	0.273355	17.1295	-1.81411	0.641802819	-0.772659895
MMP9 84kDa	CON Post - AE Post	0.828055	-1.3351	1.563219	24.03161	-0.85407	0.641802819	-2.080236983
MMP2 72kDa	CON Pre - AE Pre	0.954613	-3.45665	6.692663	36.43455	-0.51648	12.85985339	-0.268794165
MMP2 72kDa	CON Pre - CON Post	0.99919	-0.8077	6.22025	19.53295	-0.12985	12.85985339	-0.062807614
MMP2 72kDa	CON Pre - AE Post	0.403291	-11.1012	7.03622	37.38521	-1.57773	12.85985339	-0.863247994
MMP2 72kDa	AE Pre - CON Post	0.978663	2.648957	6.692663	36.43455	0.3958	12.85985339	0.205986552
MMP2 72kDa	AE Pre - AE Post	0.493095	-7.64459	5.3237	19.53628	-1.43595	12.85985339	-0.594453829
MMP2 72kDa	CON Post - AE Post	0.469459	-10.2935	7.03622	37.38521	-1.46294	12.85985339	-0.80044038
MMP2 64kDa	CON Pre - AE Pre	0.840823	-1.37371	1.659765	24.17126	-0.82765	0.908188503	-1.512580993
MMP2 64kDa	CON Pre - CON Post	0.592363	-0.57613	0.453228	17.2126	-1.27118	0.908188503	-0.634375052
MMP2 64kDa	CON Pre - AE Post	0.545235	-2.24537	1.671587	24.81103	-1.34326	0.908188503	-2.472362167
MMP2 64kDa	AE Pre - CON Post	0.96268	0.797577	1.659765	24.17126	0.480536	0.908188503	0.878205942
MMP2 64kDa	AE Pre - AE Post	0.147796	-0.87166	0.386466	17.22963	-2.25547	0.908188503	-0.959781174
MMP2 64kDa	CON Post - AE Post	0.751572	-1.66924	1.671587	24.81103	-0.9986	0.908188503	-1.837987115
ADAM10 60kDa	CON Pre - AE Pre	0.354245	-1.89805	1.133014	29.59578	-1.67522	1.444041807	-1.314399565
ADAM10 60kDa	CON Pre - CON Post	0.999337	-0.08654	0.713444	18.15881	-0.1213	1.444041807	-0.059931388
ADAM10 60kDa	CON Pre - AE Post	0.086078	-2.88453	1.172708	31.99279	-2.45972	1.444041807	-1.997540074
ADAM10 60kDa	AE Pre - CON Post	0.394712	1.811504	1.133014	29.59578	1.598837	1.444041807	1.254468177
ADAM10 60kDa	AE Pre - AE Post	0.391013	-0.98648	0.607912	18.32088	-1.62274	1.444041807	-0.683140509
ADAM10 60kDa	CON Post - AE Post	0.100301	-2.79799	1.172708	31.99279	-2.38592	1.444041807	-1.937608686
ADAM10 100kDa	CON Pre - AE Pre	0.66662	-0.5065	0.442883	25.42243	-1.14364	0.34637944	-1.462261232
ADAM10 100kDa	CON Pre - CON Post	0.891844	0.122442	0.172491	17.43524	0.709847	0.34637944	0.35349076
ADAM10 100kDa	CON Pre - AE Post	0.61632	-0.55068	0.449194	26.65402	-1.22593	0.34637944	-1.589815898
ADAM10 100kDa	AE Pre - CON Post	0.498869	0.628939	0.442883	25.42243	1.420103	0.34637944	1.815751992
ADAM10 100kDa	AE Pre - AE Post	0.990241	-0.04418	0.147062	17.47623	-0.30043	0.34637944	-0.127554666
ADAM10 100kDa	CON Post - AE Post	0.452429	-0.67312	0.449194	26.65402	-1.49851	0.34637944	-1.943306658
TLR4	CON Pre - AE Pre	0.574177	0.43347	0.335567	35.00424	1.291755	0.581839662	0.744998816
TLR4	CON Pre - CON Post	0.565026	0.373107	0.283758	19.12734	1.314879	0.581839662	0.641254022
TLR4	CON Pre - AE Post	0.055471	0.937698	0.354603	36.99546	2.644361	0.581839662	1.611609721
TLR4	AE Pre - CON Post	0.997893	-0.06036	0.335567	35.00424	-0.17988	0.581839662	-0.103744795
TLR4	AE Pre - AE Post	0.191528	0.504229	0.241406	19.5483	2.088719	0.581839662	0.866610904

TLR4	CON Post - AE Post	0.395396	0.564591	0.354603	36.99546	1.592179	0.581839662	0.970355699
TLR2	CON Pre - AE Pre	0.688544	-1.54091	1.391539	24.20782	-1.10734	0.773062176	-1.99325715
TLR2	CON Pre - CON Post	0.911049	0.254099	0.38577	17.21917	0.65868	0.773062176	0.328691613
TLR2	CON Pre - AE Post	0.498061	-1.99316	1.401749	24.86629	-1.42191	0.773062176	-2.578262116
TLR2	AE Pre - CON Post	0.577852	1.795011	1.391539	24.20782	1.289947	0.773062176	2.321948763
TLR2	AE Pre - AE Post	0.530718	-0.45225	0.328944	17.23681	-1.37484	0.773062176	-0.585004965
TLR2	CON Post - AE Post	0.39516	-2.24726	1.401749	24.86629	-1.60318	0.773062176	-2.906953729
RAGE	CON Pre - AE Pre	0.953231	-19.8375	38.03658	29.41676	-0.52154	47.82822019	-0.414765108
RAGE	CON Pre - CON Post	0.890813	-16.8445	23.63873	18.12798	-0.71258	47.82822019	-0.352187485
RAGE	CON Pre - AE Post	0.97154	17.20637	39.33835	31.79078	0.437394	47.82822019	0.35975353
RAGE	AE Pre - CON Post	0.999821	2.992976	38.03658	29.41676	0.078687	47.82822019	0.062577623
RAGE	AE Pre - AE Post	0.287793	37.04385	20.14274	18.28356	1.839067	47.82822019	0.774518639
RAGE	CON Post - AE Post	0.822412	34.05087	39.33835	31.79078	0.86559	47.82822019	0.711941015
TIMP1:MMP9 92	CON Pre - AE Pre	0.590628	125.8534	99.20264	24.90045	1.268649	68.88819096	1.826922024
TIMP1:MMP9 92	CON Pre - CON Post	0.988894	10.78212	34.33605	17.34285	0.314018	68.88819096	0.15651626
TIMP1:MMP9 92	CON Pre - AE Post	0.635471	119.8757	100.327	25.89749	1.19485	68.88819096	1.74014911
TIMP1:MMP9 92	AE Pre - CON Post	0.656854	-115.071	99.20264	24.90045	-1.15996	68.88819096	-1.670405764
TIMP1:MMP9 92	AE Pre - AE Post	0.99687	-5.97763	29.27596	17.37313	-0.20418	68.88819096	-0.086772914
TIMP1:MMP9 92	CON Post - AE Post	0.700173	109.0936	100.327	25.89749	1.087381	68.88819096	1.58363285
TIMP1:MMP9 84	CON Pre - AE Pre	0.752017	86.68352	86.87362	24.72538	0.997812	57.53023958	1.506747081
TIMP1:MMP9 84	CON Pre - CON Post	0.286909	-52.924	28.68346	17.31171	-1.8451	57.53023958	-0.91993322
TIMP1:MMP9 84	CON Pre - AE Post	0.613119	108.0944	87.77167	25.63973	1.231541	57.53023958	1.87891498
TIMP1:MMP9 84	AE Pre - CON Post	0.393196	-139.607	86.87362	24.72538	-1.60702	57.53023958	-2.426680301
TIMP1:MMP9 84	AE Pre - AE Post	0.817389	21.41091	24.45685	17.33863	0.875456	57.53023958	0.3721679
TIMP1:MMP9 84	CON Post - AE Post	0.280872	161.0184	87.77167	25.63973	1.834515	57.53023958	2.798848201
TIMP1:MMP2 72	CON Pre - AE Pre	0.117256	36.92621	15.86293	26.32898	2.327831	16.34775474	2.258794126
TIMP1:MMP2 72	CON Pre - CON Post	0.517034	11.33612	8.11235	17.77021	1.397391	16.34775474	0.693436173
TIMP1:MMP2 72	CON Pre - AE Post	0.101757	38.70165	16.16853	27.80698	2.393641	16.34775474	2.367398324
TIMP1:MMP2 72	AE Pre - CON Post	0.388797	-25.5901	15.86293	26.32898	-1.6132	16.34775474	-1.565357953
TIMP1:MMP2 72	AE Pre - AE Post	0.993875	1.775435	6.925829	17.6915	0.25635	16.34775474	0.108604198
TIMP1:MMP2 72	CON Post - AE Post	0.3465	27.36552	16.16853	27.80698	1.692518	16.34775474	1.673962151
TIMP1:MMP2 64	CON Pre - AE Pre	0.152891	49.20313	22.48846	26.28752	2.187928	20.41538485	2.410100529
TIMP1:MMP2 64	CON Pre - CON Post	0.606114	12.6671	10.1509	17.58704	1.24788	20.41538485	0.620468379
TIMP1:MMP2 64	CON Pre - AE Post	0.118286	53.09924	22.91384	27.86649	2.317344	20.41538485	2.600942538
TIMP1:MMP2 64	AE Pre - CON Post	0.382707	-36.536	22.48846	26.28752	-1.62466	20.41538485	-1.78963215
TIMP1:MMP2 64	AE Pre - AE Post	0.968715	3.896113	8.653588	17.64797	0.450231	20.41538485	0.190842009
TIMP1:MMP2 64	CON Post - AE Post	0.311156	40.43214	22.91384	27.86649	1.76453	20.41538485	1.980474158
TIMP1:ADAM10 60	CON Pre - AE Pre	0.032771	45.22116	15.56574	29.93711	2.905172	20.3370013	2.223590187
TIMP1:ADAM10 60	CON Pre - CON Post	0.999404	-1.17508	10.0406	18.21764	-0.11703	20.3370013	-0.057780471
TIMP1:ADAM10 60	CON Pre - AE Post	0.046144	44.25968	16.13481	32.37119	2.743118	20.3370013	2.176312992
TIMP1:ADAM10 60	AE Pre - CON Post	0.027465	-46.3962	15.56574	29.93711	-2.98066	20.3370013	-2.281370658
TIMP1:ADAM10 60	AE Pre - AE Post	0.999473	-0.96148	8.554848	18.39241	-0.11239	20.3370013	-0.047277195
TIMP1:ADAM10 60	CON Post - AE Post	0.039039	45.43476	16.13481	32.37119	2.815947	20.3370013	2.234093463
TIMP1:ADAM10 100	CON Pre - AE Pre	0.172581	65.60109	31.04776	29.39931	2.112909	38.98821773	1.682587439
TIMP1:ADAM10 100	CON Pre - CON Post	0.318295	-34.1329	19.27032	18.12498	-1.77127	38.98821773	-0.875467171

TIMP1:ADAM10 100	CON Pre - AE Post	0.284349	58.3391	32.10789	31.77095	1.816971	38.98821773	1.496326452
TIMP1:ADAM10 100	AE Pre - CON Post	0.015908	-99.734	31.04776	29.39931	-3.21228	38.98821773	-2.55805461
TIMP1:ADAM10 100	AE Pre - AE Post	0.970287	-7.26198	16.42043	18.27993	-0.44225	38.98821773	-0.186260987
TIMP1:ADAM10 100	CON Post - AE Post	0.033875	92.47201	32.10789	31.77095	2.88004	38.98821773	2.371793623
TIMP3:MMP9 92	CON Pre - AE Pre	0.872793	0.675399	0.892094	25.66943	0.757093	0.731619218	0.923155865
TIMP3:MMP9 92	CON Pre - CON Post	0.997814	-0.06589	0.364175	17.47874	-0.18093	0.731619218	-0.090062613
TIMP3:MMP9 92	CON Pre - AE Post	0.694664	0.993414	0.906014	27.00555	1.096467	0.731619218	1.357829255
TIMP3:MMP9 92	AE Pre - CON Post	0.839278	-0.74129	0.892094	25.66943	-0.83095	0.731619218	-1.013218478
TIMP3:MMP9 92	AE Pre - AE Post	0.737765	0.318015	0.31048	17.52514	1.02427	0.731619218	0.43467339
TIMP3:MMP9 92	CON Post - AE Post	0.650891	1.059306	0.906014	27.00555	1.169193	0.731619218	1.447891868
TIMP3:MMP9 84	CON Pre - AE Pre	0.895328	0.503023	0.716555	25.17467	0.702002	0.53158839	0.946263299
TIMP3:MMP9 84	CON Pre - CON Post	0.400735	-0.42546	0.264835	17.39147	-1.60652	0.53158839	-0.800361882
TIMP3:MMP9 84	CON Pre - AE Post	0.505772	1.021801	0.725781	26.29721	1.407864	0.53158839	1.92216554
TIMP3:MMP9 84	AE Pre - CON Post	0.573986	-0.92849	0.716555	25.17467	-1.29576	0.53158839	-1.746625181
TIMP3:MMP9 84	AE Pre - AE Post	0.136922	0.518778	0.2258	17.42725	2.297514	0.53158839	0.975902241
TIMP3:MMP9 84	CON Post - AE Post	0.21575	1.447264	0.725781	26.29721	1.994078	0.53158839	2.722527422
TIMP3:MMP2 72	CON Pre - AE Pre	0.131077	0.267659	0.118638	30.50777	2.256104	0.170017057	1.574309161
TIMP3:MMP2 72	CON Pre - CON Post	0.977385	0.033615	0.083643	18.47007	0.401884	0.170017057	0.197714037
TIMP3:MMP2 72	CON Pre - AE Post	0.021463	0.376299	0.122704	32.44948	3.066724	0.170017057	2.21330385
TIMP3:MMP2 72	AE Pre - CON Post	0.220335	-0.23404	0.118638	30.50777	-1.97277	0.170017057	-1.376595124
TIMP3:MMP2 72	AE Pre - AE Post	0.446521	0.10864	0.071489	18.37805	1.519666	0.170017057	0.638994689
TIMP3:MMP2 72	CON Post - AE Post	0.041149	0.342685	0.122704	32.44948	2.792774	0.170017057	2.015589813
TIMP3:MMP2 64	CON Pre - AE Pre	0.345091	0.352602	0.208019	28.01079	1.695046	0.231827814	1.520966216
TIMP3:MMP2 64	CON Pre - CON Post	0.963905	0.054431	0.114899	17.88585	0.473725	0.231827814	0.234789021
TIMP3:MMP2 64	CON Pre - AE Post	0.117029	0.494895	0.213767	30.11922	2.31512	0.231827814	2.134753485
TIMP3:MMP2 64	AE Pre - CON Post	0.48989	-0.29817	0.208019	28.01079	-1.43338	0.231827814	-1.286177195
TIMP3:MMP2 64	AE Pre - AE Post	0.484576	0.142293	0.097929	17.99455	1.453027	0.231827814	0.613787269
TIMP3:MMP2 64	CON Post - AE Post	0.189151	0.440465	0.213767	30.11922	2.060493	0.231827814	1.899964464
TIMP3:ADAM10 60	CON Pre - AE Pre	0.283139	0.310671	0.170821	32.52921	1.818699	0.261690556	1.187170546
TIMP3:ADAM10 60	CON Pre - CON Post	0.704158	-0.13904	0.128453	18.67016	-1.08244	0.261690556	-0.531326846
TIMP3:ADAM10 60	CON Pre - AE Post	0.091807	0.432988	0.17892	34.96167	2.420006	0.261690556	1.654581422
TIMP3:ADAM10 60	AE Pre - CON Post	0.059035	-0.44971	0.170821	32.52921	-2.63267	0.261690556	-1.718497392
TIMP3:ADAM10 60	AE Pre - AE Post	0.683048	0.122317	0.109379	18.9562	1.118287	0.261690556	0.467410876
TIMP3:ADAM10 60	CON Post - AE Post	0.014895	0.572032	0.17892	34.96167	3.19713	0.261690556	2.185908268
TIMP3:ADAM10 100	CON Pre - AE Pre	0.63081	0.403489	0.33589	30.12438	1.201252	0.444648641	0.90743308
TIMP3:ADAM10 100	CON Pre - CON Post	0.105359	-0.5344	0.219441	18.24996	-2.43527	0.444648641	-1.201844629
TIMP3:ADAM10 100	CON Pre - AE Post	0.401435	0.551988	0.348448	32.57505	1.584134	0.444648641	1.241402804
TIMP3:ADAM10 100	AE Pre - CON Post	0.042357	-0.93789	0.33589	30.12438	-2.79224	0.444648641	-2.109277709
TIMP3:ADAM10 100	AE Pre - AE Post	0.856126	0.148499	0.186963	18.43187	0.79427	0.444648641	0.333969724
TIMP3:ADAM10 100	CON Post - AE Post	0.018895	1.086387	0.348448	32.57505	3.117789	0.444648641	2.443247433

**Outcome**	**contrast**	**Group**	**estimate**	**SE**	**df**	**t.ratio**	**p.value**	**Model_Sigma**	**imate_ModelSigma**
sRAGE	PreTraining_AcuteEffect	CON	-115.267	48.44157	78.00169	-2.37951	0.019779221	123.5022562	-0.93332
sRAGE	PostTraining_AcuteEffect	CON	-132.836	48.44157	78.00169	-2.7422	0.007566054	123.5022562	-1.07558
sRAGE	ChangeInAcuteEffect_PostMinu	CON	-17.5693	68.50673	78.00169	-0.25646	0.798270076	123.5022562	-0.14226

sRAGE	PreTraining_AcuteEffect	AE	-96.6483	40.06942	78.00169	-2.41202	0.018213876	123.5022562	-0.78256
sRAGE	PostTraining_AcuteEffect	AE	-67.4841	48.44157	78.00169	-1.3931	0.167546157	123.5022562	-0.54642
sRAGE	ChangeInAcuteEffect_PostMinu	AE	29.16416	62.86608	78.00169	0.463909	0.644004774	123.5022562	0.236143
sTLR4	PreTraining_AcuteEffect	CON	-0.01294	0.153886	68.01829	-0.08409	0.933228331	0.370466098	-0.03493
sTLR4	PostTraining_AcuteEffect	CON	0.033937	0.153886	68.01829	0.220532	0.826117116	0.370466098	0.091606
sTLR4	ChangeInAcuteEffect_PostMinu	CON	0.046878	0.218338	68.0235	0.214703	0.83064157	0.370466098	0.126537
sTLR4	PreTraining_AcuteEffect	AE	0.097914	0.131685	68.07016	0.743545	0.459710504	0.370466098	0.264299
sTLR4	PostTraining_AcuteEffect	AE	0.064891	0.153903	68.01864	0.421637	0.674619261	0.370466098	0.175161
sTLR4	ChangeInAcuteEffect_PostMinu	AE	-0.03302	0.203415	68.0474	-0.16234	0.871518308	0.370466098	-0.08914

Outcome	contrast	Group	estimate	SE	df	t.ratio	p.value	Model_Sigma	imate_ModelSigma
sRAGE	TrainingEffect_PreAcute	CON	-16.1957	48.44157	78.00169	-0.33433	0.739024982	123.5022562	-0.13114
sRAGE	TrainingEffect_PostAcute	CON	-33.765	48.44157	78.00169	-0.69702	0.487859715	123.5022562	-0.2734
sRAGE	DifferenceInTrainingEffect_Post	CON	-17.5693	68.50673	78.00169	-0.25646	0.798270076	123.5022562	-0.14226
sRAGE	TrainingEffect_PreAcute	AE	212.9831	46.42882	78.49667	4.587305	1.67494E-05	123.5022562	1.724528
sRAGE	TrainingEffect_PostAcute	AE	242.1473	46.42882	78.49667	5.215453	1.45224E-06	123.5022562	1.960671
sRAGE	DifferenceInTrainingEffect_Post	AE	29.16416	62.86608	78.00169	0.463909	0.644004774	123.5022562	0.236143
sTLR4	TrainingEffect_PreAcute	CON	0.109615	0.145309	68.00014	0.754362	0.453236489	0.370466098	0.295885
sTLR4	TrainingEffect_PostAcute	CON	0.156493	0.162963	68.04207	0.960298	0.340305161	0.370466098	0.422422
sTLR4	DifferenceInTrainingEffect_Post	CON	0.046878	0.218338	68.0235	0.214703	0.83064157	0.370466098	0.126537
sTLR4	TrainingEffect_PreAcute	AE	-0.18313	0.140399	68.11974	-1.30432	0.19651296	0.370466098	-0.49431
sTLR4	TrainingEffect_PostAcute	AE	-0.21615	0.16001	68.15065	-1.35084	0.181213779	0.370466098	-0.58345
sTLR4	DifferenceInTrainingEffect_Post	AE	-0.03302	0.203415	68.0474	-0.16234	0.871518308	0.370466098	-0.08914

Supplemental file - correlation matrix

Variable_1	Variable_2	Spearman_ı	p_value	q_value	stars	gnificant_p(
Fasting Insulin	HOMA-IR	0.951	<0.001	<0.001	***	YES
NEFA	NEFA niAUC	-0.931	<0.001	<0.001	***	YES
Matsuda	HOMA-IR	-0.81	<0.001	<0.001	***	YES
Fasting Insulin	Matsuda	-0.739	<0.001	0.004	***	YES
RAGE	TLR4	0.781	<0.001	0.006	***	YES
ADAM10 (60)	ADAM10 (100)	0.76	<0.001	0.011	***	YES
ADAM10 (60)	MMP2 (64)	0.681	0.001	0.078	**	YES
NEFA	sTLR4	0.597	0.002	0.102	**	YES
ADAM10 (100)	MMP9 (92)	0.651	0.003	0.102	**	YES
ADAM10 (60)	MMP9 (92)	0.646	0.003	0.102	**	YES
TIMP1	NEFA niAUC	0.776	0.003	0.102	**	YES
Fasting Insulin	C-Peptide	0.579	0.003	0.102	**	YES
Insulin niAUC	C-Peptide niAUC	0.65	0.005	0.149	**	YES
HOMA-IR	C-Peptide	0.55	0.005	0.157	**	YES
NEFA	TIMP1	-0.616	0.006	0.175	**	YES
BMI	Fat Mass	0.576	0.008	0.200	**	YES
MMP2 (64)	MMP9 (92)	0.584	0.009	0.206	**	YES
Fat Mass	NEFA	0.543	0.013	0.297	*	YES
sTLR4	MMP2 (64)	0.546	0.016	0.297	*	YES
MMP2 (64)	TLR2	0.546	0.016	0.297	*	YES
BMI	HOMA-IR	0.508	0.016	0.297	*	YES
sTLR4	TIMP1	-0.544	0.016	0.297	*	YES
BMI	Matsuda	-0.499	0.018	0.320	*	YES
Glucose	hsCRP	0.465	0.019	0.320	*	YES
sRAGE	sTLR4	-0.454	0.020	0.320	*	YES
VO2	sRAGE	0.451	0.024	0.360	*	YES
sTLR4	NEFA niAUC	-0.544	0.024	0.360	*	YES
Matsuda	C-Peptide	-0.446	0.029	0.419	*	YES
TIMP1	TIMP3	0.495	0.031	0.438	*	YES
sRAGE	C-Peptide niAUC	0.515	0.035	0.457	*	YES
hsCRP	MMP9 (84)	-0.486	0.035	0.457	*	YES
Fat Mass	MMP9 (84)	-0.526	0.036	0.459	*	YES
Glucose	ADAM10 (60)	-0.478	0.039	0.475	*	YES
BMI	Glucose	0.43	0.040	0.476	*	YES
Fat Mass	hsCRP	0.449	0.041	0.476	*	YES
Fat Mass	NEFA niAUC	-0.547	0.043	0.483	*	YES
Lean Mass	MMP2 (72)	-0.5	0.049	0.533	*	YES
NEFA	sRAGE	-0.399	0.053	0.538		NO
ADAM10 (100)	TIMP3	-0.447	0.055	0.538		NO
HOMA-IR	Fructosamine	-0.395	0.056	0.538		NO
HOMA-IR	MMP2 (72)	-0.455	0.058	0.538		NO
Lean Mass	sRAGE	0.419	0.058	0.538		NO
TLR4	C-Peptide niAUC	-0.559	0.059	0.538		NO
Fasting Insulin	Fructosamine	-0.386	0.063	0.538		NO
ADAM10 (100)	TIMP1	-0.433	0.064	0.538		NO
Fasting Insulin	MMP9 (84)	-0.445	0.064	0.538		NO
sRAGE	RAGE	-0.43	0.066	0.538		NO

ADAM10 (100)	MMP2 (64)	0.43	0.066	0.538	NO
Lean Mass	Fructosamine	-0.407	0.067	0.538	NO
MMP9 (92)	Insulin niAUC	0.441	0.067	0.538	NO
Glucose niAUC	C-Peptide niAUC	0.453	0.068	0.538	NO
VO2	Lean Mass	0.403	0.070	0.544	NO
sRAGE	ADAM10 (60)	0.421	0.073	0.544	NO
VO2	C-Peptide niAUC	0.446	0.073	0.544	NO
sRAGE	Fructosamine	-0.357	0.074	0.544	NO
Fat Mass	sTLR4	0.395	0.077	0.555	NO
VO2	TIMP3	-0.414	0.078	0.556	NO
HOMA-IR	MMP9 (84)	-0.424	0.079	0.556	NO
NEFA	TLR2	-0.418	0.084	0.580	NO
ADAM10 (60)	TIMP1	-0.402	0.088	0.585	NO
C-Peptide	C-Peptide niAUC	-0.424	0.090	0.585	NO
BMI	Fasting Insulin	0.368	0.092	0.585	NO
sRAGE	TLR4	-0.395	0.094	0.585	NO
VO2	TLR4	-0.391	0.098	0.585	NO
sTLR4	ADAM10 (100)	0.391	0.098	0.585	NO
Fat Mass	Matsuda	-0.38	0.098	0.585	NO
BMI	MMP2 (72)	-0.389	0.099	0.585	NO
Glucose	MMP2 (72)	-0.388	0.101	0.585	NO
VO2	RAGE	-0.386	0.103	0.585	NO
Lean Mass	HOMA-IR	0.374	0.104	0.585	NO
ADAM10 (60)	TIMP3	-0.384	0.104	0.585	NO
MMP9 (84)	MMP9 (92)	0.384	0.104	0.585	NO
Fat Mass	sRAGE	-0.364	0.105	0.585	NO
sTLR4	ADAM10 (60)	0.379	0.110	0.601	NO
Fructosamine	Insulin niAUC	0.331	0.115	0.613	NO
Matsuda	MMP2 (72)	0.385	0.115	0.613	NO
RAGE	TIMP3	0.372	0.117	0.616	NO
MMP2 (72)	MMP9 (92)	-0.367	0.123	0.634	NO
C-Peptide	MMP2 (72)	-0.377	0.123	0.634	NO
Lean Mass	Fasting Insulin	0.353	0.126	0.634	NO
Glucose	Fructosamine	0.313	0.127	0.634	NO
Fat Mass	HOMA-IR	0.352	0.128	0.634	NO
Fat Mass	Glucose niAUC	0.339	0.133	0.650	NO
Fasting Insulin	MMP2 (72)	-0.362	0.140	0.675	NO
Matsuda	TLR2	0.36	0.142	0.679	NO
Fructosamine	ADAM10 (60)	-0.346	0.147	0.690	NO
Matsuda	hsCRP	-0.304	0.148	0.690	NO
RAGE	MMP2 (72)	-0.34	0.154	0.690	NO
MMP9 (84)	Glucose niAUC	-0.34	0.154	0.690	NO
Fasting Insulin	Insulin niAUC	-0.3	0.154	0.690	NO
Matsuda	MMP9 (84)	0.35	0.155	0.690	NO
VO2	Insulin niAUC	0.298	0.157	0.692	NO
BMI	C-Peptide niAUC	0.379	0.164	0.716	NO
VO2	TLR2	0.33	0.168	0.725	NO
sTLR4	MMP9 (92)	0.328	0.170	0.728	NO

Lean Mass	Insulin niAUC	-0.317	0.173	0.731	NO
C-Peptide	TLR2	-0.329	0.182	0.755	NO
RAGE	sTLR4	0.319	0.183	0.755	NO
Glucose	C-Peptide	0.281	0.184	0.755	NO
C-Peptide	TLR4	0.321	0.194	0.771	NO
ADAM10 (60)	TLR2	0.311	0.196	0.771	NO
VO2	MMP9 (92)	0.309	0.198	0.771	NO
RAGE	NEFA niAUC	-0.399	0.199	0.771	NO
MMP2 (64)	C-Peptide niAUC	0.399	0.199	0.771	NO
MMP2 (72)	NEFA niAUC	0.399	0.199	0.771	NO
Glucose	HOMA-IR	0.268	0.205	0.784	NO
sRAGE	TIMP1	0.304	0.207	0.784	NO
NEFA	Fructosamine	0.265	0.211	0.793	NO
TIMP3	Insulin niAUC	-0.307	0.216	0.793	NO
Glucose	sRAGE	-0.256	0.218	0.793	NO
sRAGE	TLR2	0.296	0.218	0.793	NO
Insulin niAUC	NEFA niAUC	-0.314	0.220	0.793	NO
TIMP3	TLR4	0.295	0.221	0.793	NO
MMP9 (84)	NEFA niAUC	0.378	0.226	0.796	NO
VO2	ADAM10 (60)	0.291	0.226	0.796	NO
Glucose	ADAM10 (100)	-0.291	0.227	0.796	NO
sRAGE	hsCRP	-0.244	0.230	0.796	NO
Matsuda	NEFA niAUC	0.306	0.232	0.796	NO
hsCRP	Glucose niAUC	0.247	0.234	0.796	NO
Fat Mass	Glucose	0.27	0.236	0.796	NO
Glucose	MMP9 (92)	-0.284	0.238	0.796	NO
Fat Mass	Fasting Insulin	0.275	0.240	0.796	NO
ADAM10 (100)	Glucose niAUC	-0.282	0.241	0.796	NO
C-Peptide	sRAGE	-0.248	0.243	0.796	NO
ADAM10 (100)	MMP9 (84)	0.277	0.251	0.808	NO
VO2	MMP2 (72)	-0.275	0.254	0.808	NO
Glucose	Matsuda	-0.241	0.257	0.808	NO
Fasting Insulin	sTLR4	-0.235	0.269	0.808	NO
TIMP3	TLR2	-0.265	0.273	0.808	NO
sRAGE	ADAM10 (100)	0.265	0.273	0.808	NO
MMP2 (64)	MMP9 (84)	0.261	0.280	0.808	NO
Fasting Insulin	ADAM10 (100)	-0.269	0.280	0.808	NO
BMI	Insulin niAUC	0.239	0.284	0.808	NO
NEFA	MMP2 (72)	-0.265	0.287	0.808	NO
sRAGE	MMP2 (64)	0.254	0.293	0.808	NO
C-Peptide	Fructosamine	-0.223	0.295	0.808	NO
TLR4	Insulin niAUC	0.261	0.295	0.808	NO
MMP9 (92)	Glucose niAUC	-0.253	0.297	0.808	NO
RAGE	C-Peptide niAUC	-0.329	0.297	0.808	NO
ADAM10 (100)	NEFA niAUC	-0.329	0.297	0.808	NO
hsCRP	Insulin niAUC	0.222	0.298	0.808	NO
Glucose	C-Peptide niAUC	-0.267	0.299	0.808	NO
sRAGE	NEFA niAUC	0.265	0.305	0.808	NO

Lean Mass	C-Peptide	0.241	0.307	0.808	NO
hsCRP	TLR2	-0.247	0.307	0.808	NO
TLR2	NEFA niAUC	0.322	0.308	0.808	NO
TLR4	NEFA niAUC	-0.322	0.308	0.808	NO
sTLR4	TLR2	0.244	0.314	0.808	NO
HOMA-IR	ADAM10 (100)	-0.251	0.316	0.808	NO
Lean Mass	sTLR4	-0.23	0.316	0.808	NO
MMP9 (92)	C-Peptide niAUC	0.315	0.319	0.808	NO
TLR2	C-Peptide niAUC	0.315	0.319	0.808	NO
MMP9 (92)	NEFA niAUC	-0.315	0.319	0.808	NO
HOMA-IR	hsCRP	0.212	0.320	0.808	NO
Matsuda	ADAM10 (100)	0.245	0.328	0.808	NO
NEFA	RAGE	0.245	0.328	0.808	NO
Fructosamine	hsCRP	0.2	0.328	0.808	NO
sRAGE	Glucose niAUC	0.204	0.328	0.808	NO
Glucose	MMP2 (64)	-0.237	0.328	0.808	NO
Fasting Insulin	TLR2	-0.243	0.332	0.808	NO
TLR2	Glucose niAUC	-0.235	0.333	0.808	NO
sRAGE	MMP9 (92)	0.235	0.333	0.808	NO
MMP9 (84)	TIMP3	0.235	0.333	0.808	NO
Fructosamine	NEFA niAUC	-0.25	0.333	0.808	NO
Glucose	MMP9 (84)	-0.234	0.334	0.808	NO
RAGE	ADAM10 (60)	-0.233	0.336	0.808	NO
hsCRP	MMP2 (64)	-0.233	0.336	0.808	NO
NEFA	MMP9 (84)	-0.24	0.336	0.808	NO
MMP2 (72)	Insulin niAUC	-0.24	0.336	0.808	NO
sRAGE	TIMP3	-0.232	0.340	0.812	NO
HOMA-IR	Insulin niAUC	-0.203	0.342	0.813	NO
Lean Mass	Glucose niAUC	0.217	0.345	0.814	NO
Lean Mass	C-Peptide niAUC	-0.27	0.350	0.819	NO
RAGE	TIMP1	-0.226	0.351	0.819	NO
Fructosamine	MMP9 (92)	-0.226	0.353	0.819	NO
Fat Mass	Fructosamine	0.211	0.358	0.819	NO
BMI	VO2	0.201	0.359	0.819	NO
VO2	Fat Mass	0.21	0.360	0.819	NO
Matsuda	Insulin niAUC	-0.195	0.362	0.819	NO
VO2	MMP9 (84)	-0.221	0.363	0.819	NO
C-Peptide	hsCRP	0.192	0.368	0.826	NO
Matsuda	Fructosamine	0.19	0.374	0.831	NO
Lean Mass	MMP9 (84)	0.235	0.380	0.831	NO
BMI	C-Peptide	0.196	0.382	0.831	NO
BMI	TLR4	-0.212	0.383	0.831	NO
hsCRP	MMP2 (72)	0.212	0.383	0.831	NO
ADAM10 (100)	TLR2	0.212	0.383	0.831	NO
TLR4	Glucose niAUC	-0.211	0.387	0.831	NO
Fasting Insulin	hsCRP	0.183	0.393	0.831	NO
NEFA	Insulin niAUC	0.183	0.393	0.831	NO
Matsuda	TLR4	-0.214	0.395	0.831	NO

HOMA-IR	TLR2	-0.214	0.395	0.831	NO
MMP2 (64)	TIMP1	-0.207	0.395	0.831	NO
Fat Mass	C-Peptide niAUC	0.244	0.401	0.838	NO
MMP9 (84)	TIMP1	0.202	0.408	0.848	NO
Fat Mass	TLR2	-0.221	0.412	0.848	NO
BMI	MMP9 (92)	0.2	0.412	0.848	NO
RAGE	Glucose niAUC	-0.198	0.416	0.853	NO
Fat Mass	TIMP1	-0.215	0.425	0.866	NO
Glucose	Insulin niAUC	0.168	0.434	0.880	NO
Fasting Insulin	MMP2 (64)	-0.195	0.438	0.880	NO
Matsuda	TIMP1	-0.195	0.438	0.880	NO
VO2	Fructosamine	-0.162	0.440	0.881	NO
BMI	NEFA	-0.171	0.446	0.887	NO
Glucose	RAGE	0.185	0.448	0.887	NO
MMP2 (72)	TLR4	-0.182	0.455	0.896	NO
NEFA	Glucose niAUC	0.159	0.458	0.898	NO
VO2	sTLR4	-0.155	0.461	0.899	NO
Fructosamine	MMP2 (72)	0.179	0.463	0.900	NO
TIMP3	C-Peptide niAUC	-0.231	0.471	0.908	NO
Glucose	TLR2	-0.176	0.472	0.908	NO
Glucose	sTLR4	0.148	0.480	0.910	NO
sTLR4	Fructosamine	0.144	0.483	0.910	NO
Fructosamine	TIMP3	0.171	0.484	0.910	NO
ADAM10 (60)	NEFA niAUC	-0.224	0.484	0.910	NO
RAGE	Fructosamine	0.169	0.488	0.910	NO
Fructosamine	ADAM10 (100)	-0.168	0.490	0.910	NO
Matsuda	TIMP3	-0.172	0.494	0.910	NO
BMI	hsCRP	0.146	0.496	0.910	NO
Fat Mass	Insulin niAUC	0.159	0.502	0.910	NO
HOMA-IR	NEFA niAUC	-0.174	0.504	0.910	NO
BMI	Glucose niAUC	0.146	0.506	0.910	NO
sRAGE	Insulin niAUC	0.143	0.506	0.910	NO
Fasting Insulin	MMP9 (92)	-0.166	0.510	0.910	NO
ADAM10 (100)	Insulin niAUC	0.166	0.510	0.910	NO
TIMP1	Insulin niAUC	0.166	0.510	0.910	NO
MMP2 (72)	C-Peptide niAUC	-0.21	0.513	0.910	NO
Lean Mass	ADAM10 (100)	0.176	0.513	0.910	NO
MMP2 (72)	TIMP1	0.16	0.514	0.910	NO
C-Peptide	MMP2 (64)	-0.164	0.515	0.910	NO
BMI	MMP9 (84)	-0.158	0.519	0.911	NO
Fat Mass	TIMP3	0.174	0.520	0.911	NO
BMI	MMP2 (64)	0.153	0.533	0.924	NO
VO2	MMP2 (64)	0.153	0.533	0.924	NO
Fructosamine	MMP2 (64)	-0.147	0.547	0.936	NO
hsCRP	MMP9 (92)	-0.147	0.547	0.936	NO
NEFA	hsCRP	0.129	0.549	0.936	NO
MMP9 (92)	TLR4	0.146	0.552	0.936	NO
NEFA	ADAM10 (100)	0.15	0.553	0.936	NO

Fructosamine	C-Peptide niAUC	0.154	0.554	0.936	NO
sTLR4	Glucose niAUC	-0.124	0.555	0.936	NO
sTLR4	MMP2 (72)	-0.142	0.562	0.942	NO
VO2	Glucose niAUC	0.12	0.568	0.949	NO
VO2	TIMP1	0.139	0.571	0.950	NO
C-Peptide	TIMP1	0.141	0.576	0.950	NO
NEFA	TLR4	0.141	0.576	0.950	NO
Fasting Insulin	NEFA niAUC	-0.145	0.580	0.953	NO
HOMA-IR	sTLR4	-0.118	0.582	0.953	NO
VO2	ADAM10 (100)	0.133	0.586	0.953	NO
ADAM10 (60)	C-Peptide niAUC	0.175	0.587	0.953	NO
Matsuda	sTLR4	0.115	0.593	0.960	NO
TIMP1	C-Peptide niAUC	0.168	0.602	0.967	NO
sTLR4	TLR4	0.126	0.606	0.967	NO
TIMP1	Glucose niAUC	-0.126	0.606	0.967	NO
Lean Mass	NEFA	-0.122	0.609	0.967	NO
MMP2 (64)	MMP2 (72)	-0.125	0.611	0.967	NO
C-Peptide	ADAM10 (60)	-0.127	0.616	0.967	NO
C-Peptide niAUC	NEFA niAUC	-0.13	0.619	0.967	NO
hsCRP	TIMP3	0.119	0.627	0.967	NO
Fat Mass	C-Peptide	0.116	0.627	0.967	NO
Fat Mass	RAGE	0.129	0.633	0.967	NO
NEFA	ADAM10 (60)	0.117	0.645	0.967	NO
MMP9 (84)	C-Peptide niAUC	-0.147	0.649	0.967	NO
BMI	Fructosamine	-0.097	0.651	0.967	NO
Fat Mass	MMP2 (64)	0.118	0.664	0.967	NO
Insulin niAUC	Glucose niAUC	-0.093	0.665	0.967	NO
ADAM10 (60)	TLR4	-0.105	0.668	0.967	NO
HOMA-IR	ADAM10 (60)	-0.108	0.669	0.967	NO
HOMA-IR	MMP2 (64)	-0.108	0.669	0.967	NO
Fat Mass	ADAM10 (100)	-0.115	0.672	0.967	NO
VO2	NEFA niAUC	-0.11	0.673	0.967	NO
C-Peptide	TIMP3	-0.106	0.675	0.967	NO
sTLR4	hsCRP	0.086	0.677	0.967	NO
Matsuda	RAGE	-0.104	0.681	0.967	NO
Fasting Insulin	ADAM10 (60)	-0.102	0.687	0.967	NO
HOMA-IR	TIMP3	0.102	0.687	0.967	NO
Lean Mass	TIMP1	0.109	0.688	0.967	NO
Lean Mass	TLR4	-0.109	0.688	0.967	NO
Fasting Insulin	Glucose niAUC	0.086	0.689	0.967	NO
C-Peptide	ADAM10 (100)	-0.098	0.699	0.967	NO
Glucose	NEFA niAUC	0.101	0.701	0.967	NO
ADAM10 (60)	Insulin niAUC	0.096	0.705	0.967	NO
ADAM10 (60)	Glucose niAUC	0.093	0.705	0.967	NO
Fasting Insulin	TIMP1	0.094	0.711	0.967	NO
VO2	Glucose	-0.077	0.713	0.967	NO
hsCRP	TIMP1	0.089	0.716	0.967	NO
MMP2 (72)	TIMP3	0.089	0.716	0.967	NO

HOMA-IR	MMP9 (92)	-0.092	0.717	0.967	NO
HOMA-IR	TIMP1	0.092	0.717	0.967	NO
Fructosamine	TIMP1	-0.088	0.721	0.967	NO
RAGE	Insulin niAUC	0.09	0.723	0.967	NO
BMI	Lean Mass	0.084	0.724	0.967	NO
MMP2 (72)	TLR2	0.086	0.726	0.967	NO
MMP9 (92)	TIMP1	-0.086	0.726	0.967	NO
BMI	sRAGE	0.075	0.728	0.967	NO
Fasting Insulin	TIMP3	0.088	0.729	0.967	NO
TIMP1	TLR4	-0.084	0.732	0.967	NO
HOMA-IR	NEFA	-0.073	0.734	0.967	NO
sTLR4	C-Peptide niAUC	-0.088	0.736	0.967	NO
BMI	TLR2	-0.082	0.737	0.967	NO
Fasting Insulin	NEFA	-0.071	0.741	0.967	NO
RAGE	MMP2 (64)	0.081	0.743	0.967	NO
Lean Mass	MMP9 (92)	0.088	0.745	0.967	NO
NEFA	MMP9 (92)	0.082	0.748	0.967	NO
MMP9 (84)	Insulin niAUC	-0.082	0.748	0.967	NO
MMP9 (92)	TLR2	0.077	0.753	0.967	NO
Lean Mass	TLR2	-0.085	0.753	0.967	NO
Matsuda	MMP2 (64)	0.077	0.760	0.967	NO
Matsuda	Glucose niAUC	0.065	0.762	0.967	NO
RAGE	MMP9 (92)	0.074	0.764	0.967	NO
MMP2 (72)	Glucose niAUC	-0.074	0.764	0.967	NO
TLR2	Insulin niAUC	0.075	0.766	0.967	NO
Lean Mass	Glucose	0.069	0.767	0.967	NO
BMI	sTLR4	-0.063	0.768	0.967	NO
ADAM10 (60)	MMP2 (72)	0.072	0.770	0.967	NO
NEFA	MMP2 (64)	-0.073	0.773	0.967	NO
Fat Mass	ADAM10 (60)	0.076	0.778	0.967	NO
sTLR4	MMP9 (84)	0.068	0.781	0.967	NO
Glucose	Fasting Insulin	0.06	0.782	0.967	NO
Fructosamine	TLR2	-0.068	0.783	0.967	NO
BMI	TIMP1	0.065	0.792	0.967	NO
sRAGE	MMP2 (72)	-0.065	0.792	0.967	NO
MMP9 (84)	TLR2	-0.065	0.792	0.967	NO
TLR2	TLR4	-0.065	0.792	0.967	NO
Lean Mass	hsCRP	-0.06	0.797	0.967	NO
Matsuda	MMP9 (92)	-0.065	0.798	0.967	NO
C-Peptide	sTLR4	-0.055	0.799	0.967	NO
RAGE	TLR2	0.06	0.808	0.967	NO
sTLR4	Insulin niAUC	0.052	0.809	0.967	NO
MMP2 (64)	NEFA niAUC	-0.077	0.812	0.967	NO
Fat Mass	Lean Mass	0.055	0.814	0.967	NO
C-Peptide	Glucose niAUC	-0.05	0.815	0.967	NO
Fasting Insulin	C-Peptide niAUC	-0.061	0.815	0.967	NO
Glucose niAUC	NEFA niAUC	0.061	0.815	0.967	NO
Matsuda	ADAM10 (60)	0.059	0.817	0.967	NO

MMP2 (64)	Insulin niAUC	0.059	0.817	0.967	NO
Fructosamine	TLR4	0.056	0.819	0.967	NO
ADAM10 (100)	TLR4	-0.056	0.819	0.967	NO
Matsuda	C-Peptide niAUC	-0.059	0.823	0.967	NO
BMI	ADAM10 (100)	-0.054	0.825	0.967	NO
C-Peptide	NEFA niAUC	0.056	0.830	0.967	NO
RAGE	ADAM10 (100)	-0.053	0.831	0.967	NO
VO2	C-Peptide	0.045	0.834	0.967	NO
RAGE	hsCRP	0.051	0.836	0.967	NO
RAGE	MMP9 (84)	-0.051	0.836	0.967	NO
hsCRP	ADAM10 (100)	-0.051	0.836	0.967	NO
TIMP1	TLR2	-0.051	0.836	0.967	NO
VO2	HOMA-IR	0.044	0.837	0.967	NO
Fructosamine	Glucose niAUC	0.043	0.838	0.967	NO
Matsuda	sRAGE	0.043	0.840	0.967	NO
NEFA	TIMP3	-0.051	0.842	0.967	NO
C-Peptide	MMP9 (84)	0.051	0.842	0.967	NO
Lean Mass	TIMP3	0.053	0.846	0.967	NO
hsCRP	TLR4	0.047	0.847	0.967	NO
HOMA-IR	RAGE	0.049	0.848	0.967	NO
hsCRP	NEFA niAUC	0.047	0.859	0.967	NO
Glucose	TIMP3	-0.043	0.861	0.967	NO
Fructosamine	MMP9 (84)	0.043	0.861	0.967	NO
BMI	TIMP3	-0.042	0.864	0.967	NO
VO2	Fasting Insulin	0.036	0.869	0.967	NO
TIMP3	Glucose niAUC	-0.04	0.870	0.967	NO
Lean Mass	Matsuda	-0.038	0.875	0.967	NO
HOMA-IR	Glucose niAUC	0.034	0.875	0.967	NO
Fat Mass	MMP2 (72)	-0.041	0.880	0.967	NO
Fat Mass	TLR4	-0.041	0.880	0.967	NO
Lean Mass	RAGE	0.041	0.880	0.967	NO
TIMP3	NEFA niAUC	0.049	0.880	0.967	NO
HOMA-IR	TLR4	0.038	0.880	0.967	NO
HOMA-IR	C-Peptide niAUC	-0.039	0.881	0.967	NO
hsCRP	C-Peptide niAUC	-0.039	0.881	0.967	NO
MMP2 (72)	MMP9 (84)	-0.035	0.887	0.970	NO
Fasting Insulin	RAGE	-0.034	0.893	0.975	NO
sTLR4	TIMP3	-0.03	0.904	0.981	NO
Fasting Insulin	sRAGE	-0.026	0.904	0.981	NO
HOMA-IR	sRAGE	-0.023	0.913	0.987	NO
ADAM10 (100)	C-Peptide niAUC	-0.035	0.914	0.987	NO
Fat Mass	MMP9 (92)	-0.026	0.922	0.990	NO
NEFA	C-Peptide niAUC	0.025	0.926	0.990	NO
hsCRP	ADAM10 (60)	-0.023	0.926	0.990	NO
MMP9 (92)	TIMP3	-0.021	0.932	0.990	NO
sRAGE	MMP9 (84)	0.021	0.932	0.990	NO
Matsuda	NEFA	-0.018	0.933	0.990	NO
Glucose	Glucose niAUC	-0.017	0.935	0.990	NO

BMI	NEFA niAUC	0.021	0.940	0.990	NO
Lean Mass	MMP2 (64)	0.021	0.940	0.990	NO
C-Peptide	Insulin niAUC	-0.016	0.942	0.990	NO
VO2	hsCRP	-0.015	0.945	0.990	NO
Lean Mass	NEFA niAUC	0.02	0.946	0.990	NO
BMI	RAGE	-0.016	0.949	0.990	NO
ADAM10 (100)	MMP2 (72)	-0.014	0.955	0.994	NO
Lean Mass	ADAM10 (60)	-0.015	0.957	0.994	NO
Glucose	TIMP1	-0.011	0.966	0.997	NO
VO2	NEFA	-0.009	0.968	0.997	NO
BMI	ADAM10 (60)	-0.009	0.972	0.997	NO
MMP9 (84)	TLR4	-0.009	0.972	0.997	NO
Glucose	NEFA	-0.007	0.973	0.997	NO
ADAM10 (60)	MMP9 (84)	0.007	0.977	0.997	NO
MMP2 (64)	TLR4	0.007	0.977	0.997	NO
C-Peptide	NEFA	-0.004	0.984	0.999	NO
Fasting Insulin	TLR4	0.003	0.990	0.999	NO
C-Peptide	MMP9 (92)	-0.003	0.990	0.999	NO
Glucose	TLR4	-0.003	0.991	0.999	NO
MMP2 (64)	TIMP3	-0.002	0.994	0.999	NO
C-Peptide	RAGE	0.001	0.997	0.999	NO
VO2	Matsuda	0.001	0.997	0.999	NO
MMP2 (64)	Glucose niAUC	0	1.000	1.000	NO
